# Prediabetes, diabetes, and folate status among United States women of reproductive age: NHANES 2011–March 2020

**DOI:** 10.1016/j.ajcnut.2026.101193

**Published:** 2026-01-10

**Authors:** Krista S Crider, Olufunmilola Adisa, Christine M Pfeiffer, Arick Wang, Ying Zhou, Lorraine F Yeung, Kai M Bullard, Yan Ping Qi, Charles Rose, Zia Fazili, Jennifer L Williams

**Affiliations:** 1 National Center on Birth Defects and Developmental Disabilities, Centers for Disease Control and Prevention, Atlanta, GA, United States; 2 Lukos LLC, Atanta, GA, United States; 3 National Center for Environmental Health, Centers for Disease Control and Prevention, Atlanta, GA, United States; 4 National Center for Chronic Disease Prevention and Health Promotion, Centers for Disease Control and Prevention, Atlanta, GA, United States

**Keywords:** nutrition, obesity, folic acid, diabetes, prediabetes, MeFox, folate, vitamin B_12_, dietary supplements, birth defects risks

## Abstract

**Background:**

Pregestational diabetes increases the risk of adverse outcomes including congenital malformations, stillbirth, developmental disabilities, and maternal morbidity. Periconceptional glycemic control and folic acid (FA) supplementation are 2 of the most effective birth defects prevention strategies.

**Objectives:**

The objectives are to describe the proportion of and risk factors for diabetes and prediabetes and assess the association of folate status and diabetes among a nationally representative sample of nonpregnant women of reproductive age (WRA).

**Methods:**

WRA (12–49 y) from the NHANES 2011–March 2020 (*n* = 3731) were included. Diabetes status was defined by glycated hemoglobin (HbA1c) ≥6.5%, fasting plasma glucose (FPG) ≥126 mg/dL, or self-report. Prediabetes was defined as HbA1c ≥5.7 <6.5% or FPG ≥100 <126 mg/dL. The associations were assessed by multivariate regression models.

**Results:**

Among all WRA, 32.3% [95% confidence interval (CI): 30.0%, 34.7%] had prediabetes and 5.3% (95% CI: 4.4%, 6.3%) had diabetes [1.8% undiagnosed, 95% CI: 1.4%, 2.3%; 3.1% diagnosed but uncontrolled (HbA1c ≥5.7), 95% CI: 2.5, 4.0; 0.4% diagnosed but controlled (HbA1c<5.7), 95% CI: 0.2, 0.6]. The prevalence of diabetes was associated with increased age, BMI, serum pyrazino-*s*-triazine, an oxidation form of 5′-methyltetrahydrofolate (MeFox), and red blood cell (RBC) folate concentrations (all *P* < 0.0001) but not unmetabolized FA. Among WRA ≥35 y, 10.5% (95% CI: 8.5%, 12.8%) had diabetes and 40.3% (95% CI: 37.1%, 43.5%) had prediabetes. In adjusted regression models, diabetes was associated with altered folate metabolism [i.e., high (>90th %) RBC folate concentrations with lower (<400 μg/d) FA intake; adjusted odds ratio 2.28 (95% CI: 1.23, 4.24)]. Among those with diabetes, high serum MeFox and RBC folate concentrations were lower with euglycemia.

**Conclusions:**

Diabetes and prediabetes were common among WRA. Diabetes was associated with high RBC folate concentration and high MeFox despite low FA intake; however, these associations were reduced among those with good glycemic control. Screening for and preventing the progression of prediabetes, diagnosis, and glycemic control among those with diabetes has the potential to prevent adverse outcomes.

## Introduction

Pregestational diabetes and low folate status are 2 of the strongest known modifiable risk factors for birth defects [1–7]. Understanding the complex interactions of diabetes and folate status is critical for the appropriate implementation of intervention strategies and for optimal maternal and infant health. Diabetes is a chronic metabolic disease associated with long-term damage to the heart, blood vessels, eyes, kidneys, and nerves. In the United States, the number of adults diagnosed with diabetes has more than doubled in the last 20 y, and 38.4 million Americans are currently living with diabetes (11.6% of the United States population) [8]. Pregestational diabetes has been seen in ~1% to 2% of pregnancies and is associated with complications for both the mother and infant including preeclampsia, birth defects, stillbirth, premature birth, macrosomia, and adverse neurodevelopmental outcomes [6,9–12]. The number of females aged 12 to 49 y who have diabetes has increased from 3.2% to 6.0% between 2007 and 2020 [13]. Both experimental and epidemiological studies have shown associations between higher glucose concentrations during pregnancy and increased risk of birth defects among females with diabetes [6,9,10,14–18].

Folate is a key source of the 1-carbon group used in developmental gene regulation, cell signaling, DNA/RNA synthesis, and cell growth critical in early embryo and fetal development [3]. Folate is an umbrella term used to describe many vitamers (folate forms), each is linked to different outcomes [19–22]. Folic acid (FA) is the only folate form that has been shown through randomized control trials to reduce the risk of having a pregnancy affected by neural tube defects (NTDs)—major birth defects of the brain and spine leading to death or disability [23–26]. As red blood cell (RBC) folate concentrations in the mother decrease from sufficiency to deficiency, the risk of NTDs increases by 10-fold [27]. Several studies reported that FA intake may decrease the risk of a wide range of birth defects in females with diabetes, including reducing but not eliminating the risk of spina bifida [5,6]. However, other studies observed an association between diabetes and higher RBC folate concentrations [28–31], which may be associated with an increased risk of mortality [7]. It is unclear whether high folate concentrations among persons with diabetes are associated with folate intake (FA or total folate) or are an artifact of another process (i.e., a methyl-trap, where folates forms are present but cannot be used or processed properly due to an accumulation in the cell of partially processed forms such as higher tetrahydrofolate but lower 5-methyltretrahydrofolate and DNA replication can proceed but not methylation reactions) [21]. It is important to explore these critical interactions of diabetes and folate to ensure appropriate screenings and recommendations. Our study aims were *1*) to describe the proportion of and risk factors for diabetes and prediabetes, and *2*) to assess the associations between diabetes status, folate biomarker concentrations, and the modeled daily usual intake of folate by diabetes status among women of reproductive age (WRA) (females aged 12–49 y) in the United States, using the NHANES data.

## Methods

### Study population

**FIGURE 1.**
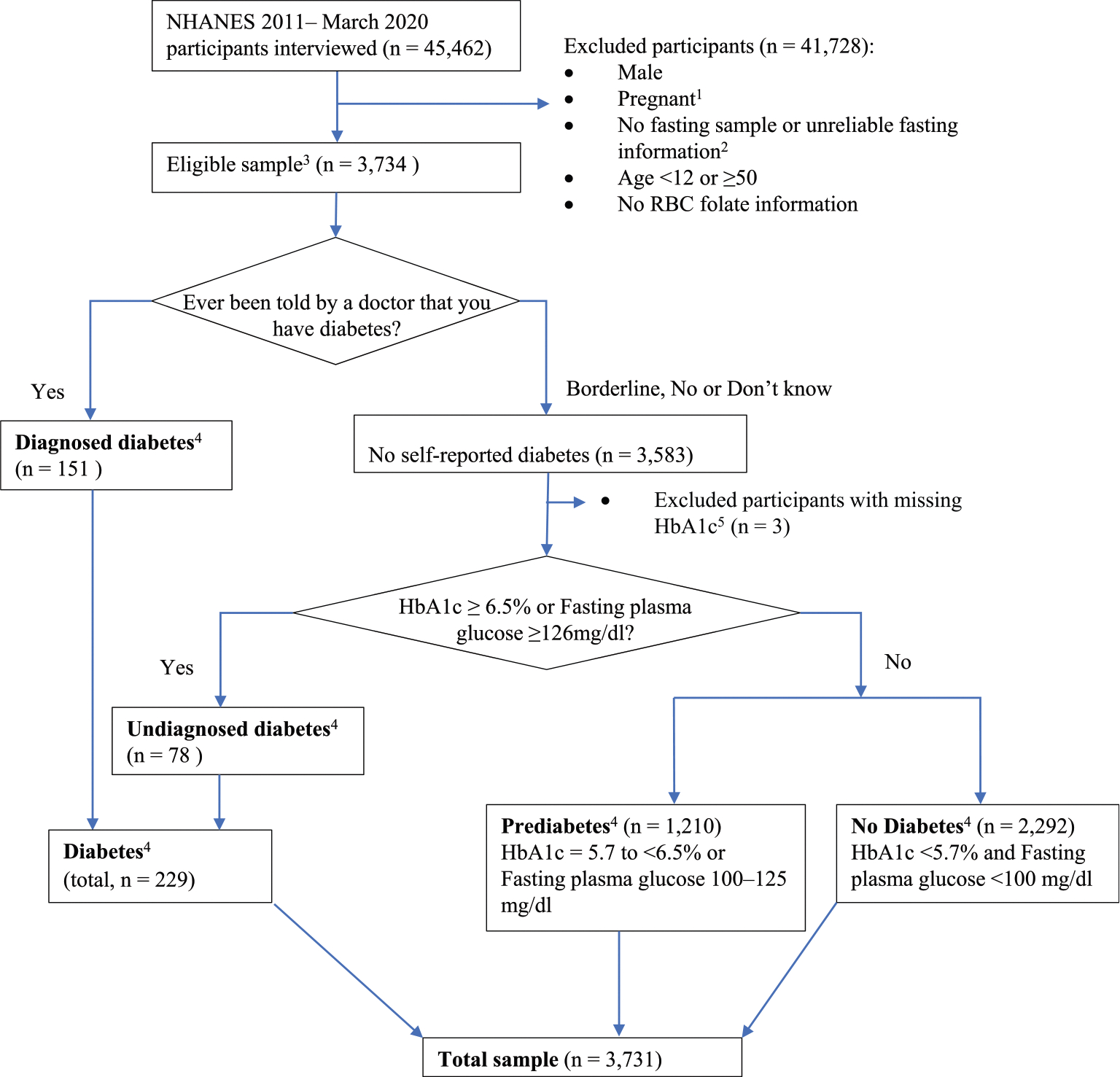
Study population and diabetes status: NHANES 2011–March 2020. ^1^Pregnant: yes, positive laboratory pregnancy test or self-reported pregnant at exam. ^2^Ineligible fasting participants: “unreasonable fasting hours + valid FPG.” ^3^Participants with eligible fasting sample. The exclusion criteria include male (*n* = 6309), pregnant (*n* = 97),^1^ age <12 or ≥50 (*n* = 2822), nonfasting (*n* = 26,289) and participants without RBC folate (*n* = 26) or with unreliable fasting information (*n* = 6185).^2^ Eligible fasting participants responded to the question “Other than during pregnancy, have you ever been told by a doctor or health professional that you have diabetes or sugar diabetes?” (DIQ010). ^4^Diabetes status was defined as diabetes, prediabetes and no diabetes (Division of Diabetes Translation, CDC), Diabetes status definition [8,9,34], Diabetes: diagnosed diabetes criteria: self-reported “Yes” to DIQ010 or undiagnosed diabetes criteria: did not self-report “Yes” to DIQ010 with HbA1c ≥6.5% or FPG ≥126 mg/dL (there are 62 participants who did not report physician-diagnosed diabetes, with FBS ≥126 mg/dL. All but 9 had HbA1c in the diabetic/prediabetic range; 4 had FBS >140 mg/dL (141–225 mg/dL) and 5 between ≥126 and <140 mg/dL). Prediabetes: did not self-report “Yes” to DIQ010 with HbA1c = 5.7 to <6.5% or FPG = 100–125 mg/dL. No diabetes: did not self-report “Yes” to DIQ010 with HbA1c <5.7% and FPG <100 mg/dL. ^5^Exclusion of participants who did not self-report “Yes” to DIQ010 without HbA1c information (*n* = 3). FBS, fasting blood sugar; FPG, fasting plasma glucose; HbA1c, glycated hemoglobin; RBC, red blood cell.

NHANES is a program of cross-sectional studies designed to assess the health and nutritional status of adults and children in the United States [32]. NHANES is a stratified multistage probability survey representative of the civilian, noninstitutionalized, United States population. Data are collected via household interviews, phone interviews, and health examinations, including fasting laboratory tests [32]. NHANES is approved by the National Center for Health Statistics Ethics Review Board, and participants provided written informed consent before participation. Our study population consisted of fasting nonpregnant WRA in NHANES 2011–March 2020. All participants self-identified as females. We excluded participants who were non-fasting, pregnant, male, with missing RBC folate or glycated hemoglobin (HbA1c), or with unreliable fasting information (Figure 1). Survey cycle response rates ranged from ~50% to 80% [33].

### Diabetes status

Respondents were classified as “diagnosed diabetes” by answering “Yes” to the question “Other than during pregnancy, have you ever been told by a doctor or health professional that you have diabetes or sugar diabetes?” Respondents who did not answer “Yes” were classified based on their HbA1c or fasting plasma glucose (FPG) as “undiagnosed diabetes” (HbA1c ≥6.5% or FPG ≥126 mg/dL), “prediabetes” (HbA1c 5.7%–<6.5% or FPG 100–125 mg/dL) or “no diabetes” (HbA1c <5.7% and FPG <100 mg/dL) (Figure 1) [8,34,35]. Diabetes control was defined by using 3 different commonly used cut points for glycemic control: HbA1c <5.7%, <6.5%, and <7.0% mg/dL.

### Covariates

We categorized age in years into 2 groups: 12 to 34 (<35 y) and 35 to 49 (≥35 y). We divided race and ethnicity based on self-reports into 5 categories: Hispanic, non-Hispanic (NH) Black, NH White, NH Asian, or NH other (including multiple races). We combined Mexican Americans and Other Hispanics into 1 Hispanic category to increase sample size and produce stable estimates [36–39]. We defined smoking exposure based on serum cotinine concentrations—a measurement that reflects recent exposure to nicotine in tobacco smoke, using an isotope dilution-HPLC. We classified participants’ smoking exposure with cotinine concentrations <10 ng/mL as nonsmokers, and ≥10 ng/mL as smokers [40,41]. BMI was calculated as weight in kilograms divided by height in meters squared, based on body measures. We defined BMI as a categorical variable with 3 levels: under/normal weight (BMI <25 kg/m^2^), overweight (25–<30 kg/m^2^), and obesity (≥30 kg/m^2^). Among Asians (NHA), BMI cut points were adjusted based on WHO criteria: underweight (BMI <18.5 kg/m^2^), normal weight (18.5≤ BMI <23 kg/m^2^), overweight (23≤ BMI <27.5 kg/m^2^), and obesity (BMI ≥27.5 kg/m^2^) [42]. Family-income-poverty ratio (FIPR) was calculated by dividing family (or individual) income by the poverty guidelines specific to the survey year and classified into 4 categories: <1 (below defined poverty threshold), 1–<2, 2–<4, and ≥4.

### Folate biomarkers

Serum folate forms were measured by isotope dilution HPLC coupled to tandem mass spectrometry (liquid chromatography coupled to tandem mass spectrometry) between 2011 and 2020 [32]. Imputed values [limit of detection (LOD) divided by √2] were used in the calculation of serum total folate (sum of 5 biologically active folate forms) if any folate form result was <LOD (*n* = 23). Whole-blood folate was measured by microbiologic assay, and RBC folate was calculated after subtracting the contribution of serum (total) folate and normalizing the whole-blood folate concentration to the hematocrit [32]. This analysis includes: serum folate, RBC folate, the ratio of RBC folate to serum folate, serum pyrazino-s-triazine derivative of 4-alpha-hydroxy-5-methyltetrahydrofolate (MeFox), and unmetabolized folic acid (UMFA). We categorized RBC folate based on the optimal blood folate concentration (OBF) of 748 nmol/L (OBF: <748 nmol/L compared with ≥748 nmol/L) [43] and ≤90th percentile compared with >90th percentile (≤1700 nmol/L compared with >1700 nmol/L). We categorized serum MeFox as ≤90th percentile compared with >90th percentile (≤2.2 nmol/L compared with >2.2 nmol/L).

### Folate usual intake

The estimated supplement use was based on 30-d supplement use data collected via NHANES household interviews [32]. For each participant, the amount of FA was summed across all supplements consumed and divided by 30 to yield the mean daily amount of supplemental nutrients. We categorized FA supplement use *1*) as “Yes” (participants who took FA-containing supplements) and “No” (non-users), and *2*) as “<400 μg/d” and “≥400 μg/d.” Three FA intake sources: enriched cereal-grain product/corn masa flour (ECGP/CMF), ready-to-eat (RTE) cereals, and FA supplements (SUP) were used to categorize 4 FA consumption groups: *1*) those whose FA intake were solely from fortified food sources (ECGP/CMF only), *2*) those who consumed both ECGP/CMF and RTE cereals (ECGP/CMF + RTE), *3*) those who consumed ECGP/CMF and took SUP (ECGP/CMF + SUP), and *4*) those who consumed ECGP/CMF, RTE cereals and SUP (ECGP/CMF + RTE + SUP). We categorized RBC folate concentration-FA intake into 4 groups: >90th percentile and ≥400 μg, >90th percentile and <400 μg, ≤90th percentile and ≥400 μg, and ≤90th percentile and <400 μg.

We assessed the usual intake of dietary food folate (i.e., natural food folates and foods fortified with FA) and FA supplements. Usual dietary folate intake was modeled using two 24-h dietary recalls. Total folate intake in micrograms (μg) dietary folate equivalents (DFE) is defined as micrograms of natural food folates intake plus 1.7 × μg of FA intake from fortified foods and/or supplements containing FA. We modeled usual total folate intake using the National Cancer Institute (NCI) method [44], accounting for race, age (continuous), BMI (continuous), FIPR (continuous), and weekday/weekend consumption. Dietary weights for day 1 of the recall were used for usual intake calculation. Population distributions (e.g., median and IQR) of usual intake were calculated using the DISTRIB macro of the NCI method by generating 100 pseudo-persons for each participant in the dataset [44].

### Statistical analysis

All analyses were conducted using SAS (version 9.4; SAS Institute) and SAS-callable SUDAAN software (version 11.0; RTI International) to account for the clustered design and probabilistic selection and nonparticipation. Analyses were replicated in R, version 4.4.0, using the *survey* package to account for complex survey design and nonparticipation [45,46]. To create combined weights from 2011 to 2020, we multiplied fasting weights by 2/9.2 (2011–2016; 3 survey cycles), and by 3.2/9.2 (2017–March 2020 prepandemic; a 3.2-y period) [47]. Forward regression was performed on FPG on NHANES data from 2011 to 2014 to ensure comparability to NHANES data from 2015 to March 2020 [48]. Weighted proportions and 95% confidence intervals (CIs) in each of the 3 diabetes status groups were estimated in the overall study population and by demographic characteristics and other potential confounders or effect modifiers, e.g., smoking exposure, BMI category, FIPR category, supplement use status, and kidney function category using estimated glomerular filtration rate (eGFR). We used natural log transformations on the biomarker concentrations (serum folate, RBC folate, RBC-to-serum folate ratio, serum MeFox, and UMFA) and estimated the geometric means via multiple linear regression, adjusting for age category, race/ethnicity, current smoking exposure, BMI (continuous), FIPR (continuous), supplement use (Yes/No), and eGFR (continuous). We assessed statistical differences in the adjusted geometric and usual intake means across groups using the Wald test. All tests were 2-tailed with an α = 0.05. Reliability of proportions was assessed using the nominal or effective sample size (<30) and degree of freedom (<8). Adjusted odds ratios (aORs) were calculated using logistic regression to determine the association between RBC folate concentration-folate intake groups and diabetes status; diabetes compared with no diabetes and RBC (>90 percentile compared with ≤90 percentile), and serum MeFox (>90 percentile compared with ≤90 percentile). Modeled covariates were retained if they produced a >10% change in effect or were included a priori. Due to cell sizes, the 90th percentile was chosen for statistical stability. Population estimates were based on estimated proportions with CIs (using the degrees of freedom), multiplied by population totals (each survey cycle contributes a quarter of the population totals) [49].

## Results

### Sample selections and demographic characteristics

The sample selection and percentages of nonpregnant WRA with diabetes and prediabetes by standard demographic characteristics are shown in Figure 1 and Table 1, respectively. Among those WRA in the fasting sample with RBC folate information in NHANES 2011–March 2020 (*n* = 3731), 5.3% (95% CI: 4.4%, 6.3%) had diabetes [(*n* = 229), with 3.5% of females (*n* = 151) self-reported having diabetes and 1.8% (95% CI: 1.4%, 2.3%) of females (*n* = 78) had undiagnosed diabetes] and 32.3% (95% CI: 30.0%, 34.7%) had prediabetes (*n* = 1210) (Table 1, Figure 1). This corresponds to 4.2 million (95% CI: 3.5, 5.0) WRA with diabetes and 25.8 million (95% CI: 24.0, 27.7) WRA with prediabetes, 1.4 million (95% CI: 1.0, 1.8) with undiagnosed diabetes in the total United States population, respectively [data not shown (DNS)]. In addition, 2% of the overall participants had HbA1c concentrations ≥6.5% and 1.7% ≥7% (DNS). Among WRA who self-reported as having been told by a doctor that they had diabetes (*n* = 151), 39 (24.9%, 95% CI: 15.0%, 34.8%) reported they were not taking medications to treat diabetes (e.g., insulin, metformin). Only 21.9% (95% CI: 7.1%, 36.8%) (*n* = 10) of these females who were not reporting any diabetes medication use, also had euglycemia (DNS). Among WRA <35 y, 1.9% (95% CI: 1.3%, 2.8%) had diabetes compared with 10.5% (95% CI: 8.5%, 12.8%) among WRA ≥35 y. Among those <25 y, only 0.8% (95% CI: 0.5%, 1.4%) had diabetes and 24.1% prediabetes (95% CI: 20.9%, 27.6%) (Table 1). Among WRA with obesity, 11.2% (95% CI: 9.2%, 13.5%) had diabetes, and 44.8% (95% CI: 41.2%, 48.4%) had prediabetes. In comparison, among WRA who were under/normal weight, only 0.7% (95% CI: 0.3%, 1.4%) had diabetes, and 20.5% (95% CI: 17.8%, 23.5%) had prediabetes (Table 1).

When looking among WRA with diabetes, 5.4% (95% CI: 3.1%, 9.1%) were <25 y whereas 77.8 % (95% CI: 69.8%, 84.2%) were ≥35 y (Supplemental Table 1). Less than a third of WRA with diabetes [29.1% (95% CI: 22.9%, 36.2%)] reported consumption of a FA-containing supplement, and only 19.0% (95% CI: 13.0%, 27.0%) reported consumption of FA supplement ≥400 μg/d (Supplemental Table 1). RBC folate and serum MeFox concentrations were more likely to be in the 90th percentage among those with diabetes (Supplemental Table 1). Among those with diagnosed diabetes, 89.3% (95% CI: 82.2%, 93.8%) were uncontrolled at the HbA1c cut point of ≥5.7%, 58.2% (95% CI: 47.8%, 67.9%) representing 2.4 million (95% CI: 1.8, 3.0) WRA, at HbA1c cocnentrations ≥6.5% and 47.7% (95% CI: 38.5%, 57.0%) at the HbA1c concentrations ≥7.0% (DNS). During the time period covered in this analysis, the proportion of diabetes and prediabetes was not significantly different between the most recent (2017–March 2020) and the earliest (2011–2012) time period [diabetes: NHANES 2017–March 2020 6.6% (95% CI: 4.8%, 8.9%) compared with NHANES 2011–2012 4.7% (95% CI: 3.3%, 6.7%), *P* = 0.16, *P* = 0.12 trend 2011–2020; prediabetes: 35.7% (95% CI: 30.5%, 41.2%) compared with 30.4% (95% CI: 25.2%, 36.1%), *P* = 0.16; *P* = 0.08 trend 2011–2020; Supplemental Figure 1A, B].

### Folate biomarkers

**FIGURE 2.**
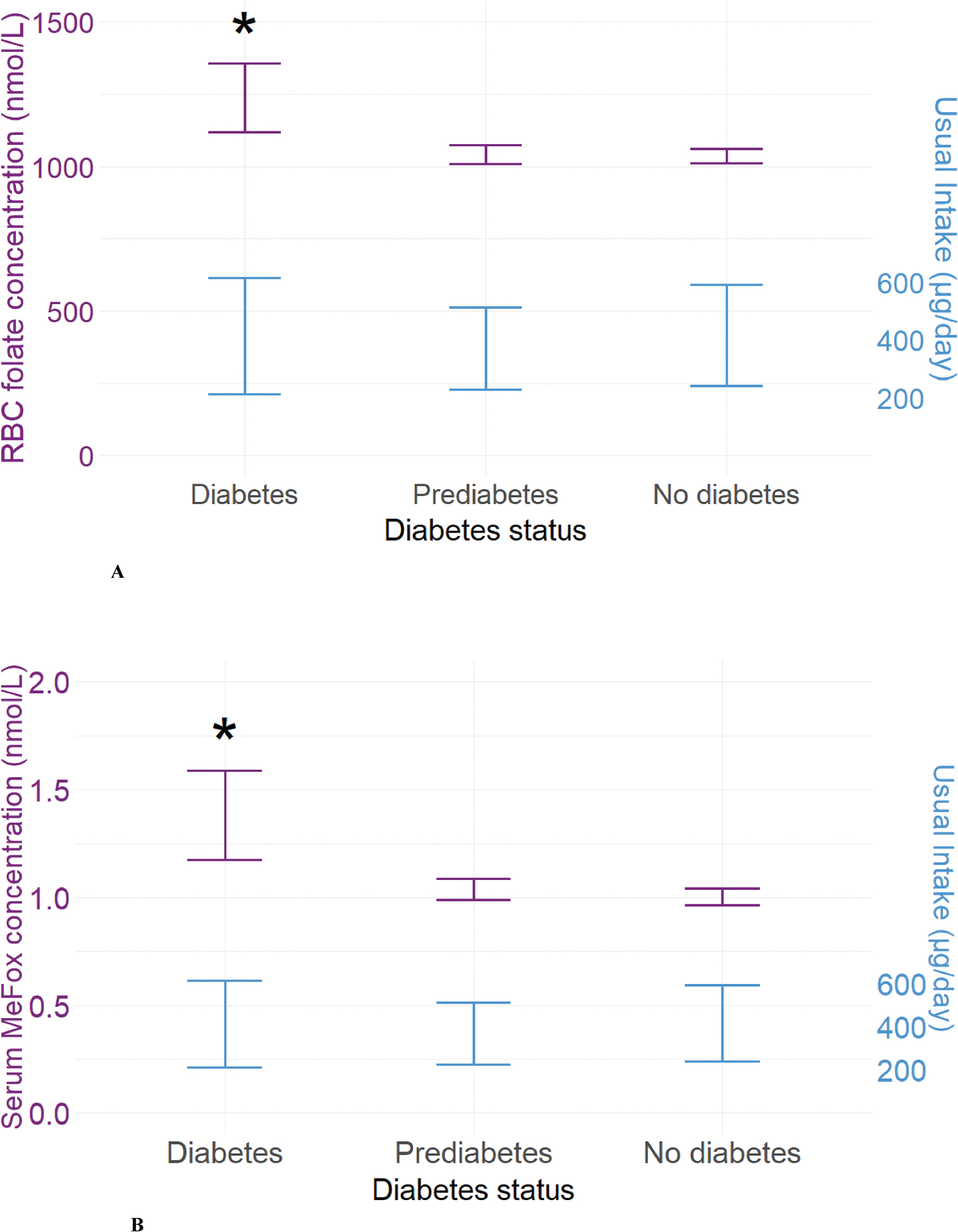
(A) Adjusted RBC folate concentrations (mean and 95% CI) and adjusted median total folate intake (IQR) among nonpregnant women of reproductive age, 12–49 y by diabetes status: NHANES 2011–March 2020. RBC folate concentrations^1^ were higher with statistically significant differences between participants with diabetes and those without diabetes, with no difference in median folic acid intake^2, 3^ (μg/d). (B) Adjusted serum MeFox concentrations (mean and 95% CI) and adjusted median total folate intake (IQR) among nonpregnant women of reproductive age, 12–49 y by diabetes status: NHANES 2011–March 2020. Serum MeFox concentration^1^ was higher with statistically significant differences between participants with diabetes and those without diabetes, with no difference in median folic acid intake^2, 3^ (μg/d). ^1^Adjusted for age, race, BMI, estimated glomerular filtration rate, family-income-poverty ratio, smoking exposure, and supplement use. ^2^Median intake and IQR accounting for the complex sampling design, adjustment for variations in day 1, day 2 measurements, and by the day of the week; statistical analyses performed using Monte Carlo simulated pseudo-persons (each participant generated 100 pseudo-persons of the participant). ^3^Adjusted for adjusted for age, race, family-income-poverty ratio, and BMI. Pink line: RBC folate and serum MeFox concentrations (nmol/L); blue line: usual intake of total folate intake from foods and supplements (μg/d). Reference: no diabetes. *P <0.05 indicates statistical significance. CI, confidence interval; MeFox, pyrazino-s-triazine derivative of 4-alpha-hydroxy-5-methyltetrahydrofolate; RBC, red blood cell.

Univariate analysis of RBC folate concentrations, serum folate, and 6 individual folate forms (serum MeFox, UMFA, 5-methyltetrahydrofolate, 5-formyltetrahydrofolate, tetrahydrofolate, 5,10-methenyltetrahydrofolate) by diabetes status is presented in Table 1. Of the 6 folate individual forms measured, we only included UMFA and MeFox, a 5-methyltetrahydrofolate (5-methylTHF) oxidation product, for further analysis based on a priori interest. Adjusted RBC folate concentrations were significantly higher among WRA with diabetes compared with WRA without diabetes in the overall study population [1232 nmol/L (95% CI: 1119, 1357) compared with 1036 nmol/L (95% CI: 1012, 1061), *P* = 0.0012] and in several subgroups, including females who were <35 y, ≥35 y, NH White, NH Black, under/normal weight and those with obesity (Supplemental Figure 2A). Adjusted serum MeFox concentrations among WRA with diabetes were significantly higher compared with those without diabetes in the overall study population [1.37 nmol/L (95% CI: 1.17, 1.59) compared with 1.00 nmol/L (95% CI: 0.96, 1.04), *P* = 0.0002], and in WRA who were ≥35 y, NH White, NH Black, Hispanic, overweight, and those with obesity (Supplemental Figure 2B). Adjusted concentrations for RBC folate and UMFA were not different in the overall study population or in any of the subgroups, or between WRA with prediabetes and those without diabetes (Figure 2A, Supplemental Figures 2B). Adjusted serum MeFox concentration and RBC-to-serum ratio were significantly higher among WRA with prediabetes compared with those without diabetes in Hispanics and the under/normal weight group, respectively (Supplemental Figure 2B, E).

Adjusted serum folate concentrations among WRA with diabetes were not significantly different from those without diabetes in the overall study population [41 nmol/L (95% CI: 35, 47) compared with 35 nmol/L (95% CI: 34, 37), *P* = 0.07], though concentrations were significantly higher among NH Black, Hispanic, and overweight WRA with diabetes compared with those without (Supplemental Figure 3A). Adjusted UMFA concentrations among WRA with diabetes were not significantly different from those without diabetes in the overall study population [2.98 nmol/L (95% CI: 1.75, 5.07) compared with 2.65 nmol/L (95% CI: 2.14, 3.28), *P* = 0.70] nor were adjusted RBC-to-serum ratios (Supplemental Figure 3B, C).

### Usual intake

RBC folate and serum MeFox concentrations were higher among those with diabetes; modeled usual total folate intake and FA intakes did not significantly differ between WRA with diabetes compared with WRA without diabetes (Figure 2A, B and Supplemental Table 2). Usual total folate intake in DFE/d was 536 (IQR: 268–891) among those with diabetes compared with 553 (IQR: 312–847) among those without diabetes [total folate in μg/d was 391 (IQR: 212–616) among those with diabetes compared with 402 (IQR: 240–593) among those without diabetes]. Usual FA intake (μg/d) was 197 (IQR: 78–384) among those with diabetes compared with 205 (IQR: 96–359) for those without diabetes (Supplemental Table 2).

### Factors of the risk of diabetes, high RBC folate, and serum MeFox concentrations

In multivariate logistic regression models, a higher risk of having diabetes (compared with no diabetes) was associated with age, BMI, serum MeFox, and belonging to a race/ethnicity group other than NH White (Table 2). Those with high folate concentrations and lower recommended FA intake were more likely to have diabetes [aOR: 2.28 (95% CI: 1.23, 4.24), *P* value: <0.05] (Table 2). Comparing WRA with diabetes to those without, there was a ~2.6-fold increased odds having high folate status (RBC folate concentration >90th percentile) adjusting for age, race, BMI, and FA intake [aOR: 2.64 (95% CI: 1.46, 4.77), *P* value: <0.05] (Table 3). In multivariable logistic regression models, high serum MeFox concentrations (>90th percentile compared with ≤ 90th percentile) were associated with diabetes (aOR 2.09, 95% CI: 1.33, 3.28) and BMI (aOR 1.05, 95% CI: 1.03, 1.06) but not FA intake over 400 μg/d (OR 1.35 95% CI: 0.92, 1.54) (Table 4). In sensitivity analysis, we confirmed that this association of diabetes with high serum MeFox was driven by uncontrolled diabetes (aOR 2.36, 95% CI: 1.44, 3.87) or continuous HbA1c (aOR 1.30, 95% CI: 1.16, 1.46) (Supplemental Table 3A–C).

### Association of euglycemia on RBC folate and serum MeFox concentrations

**FIGURE 3.**
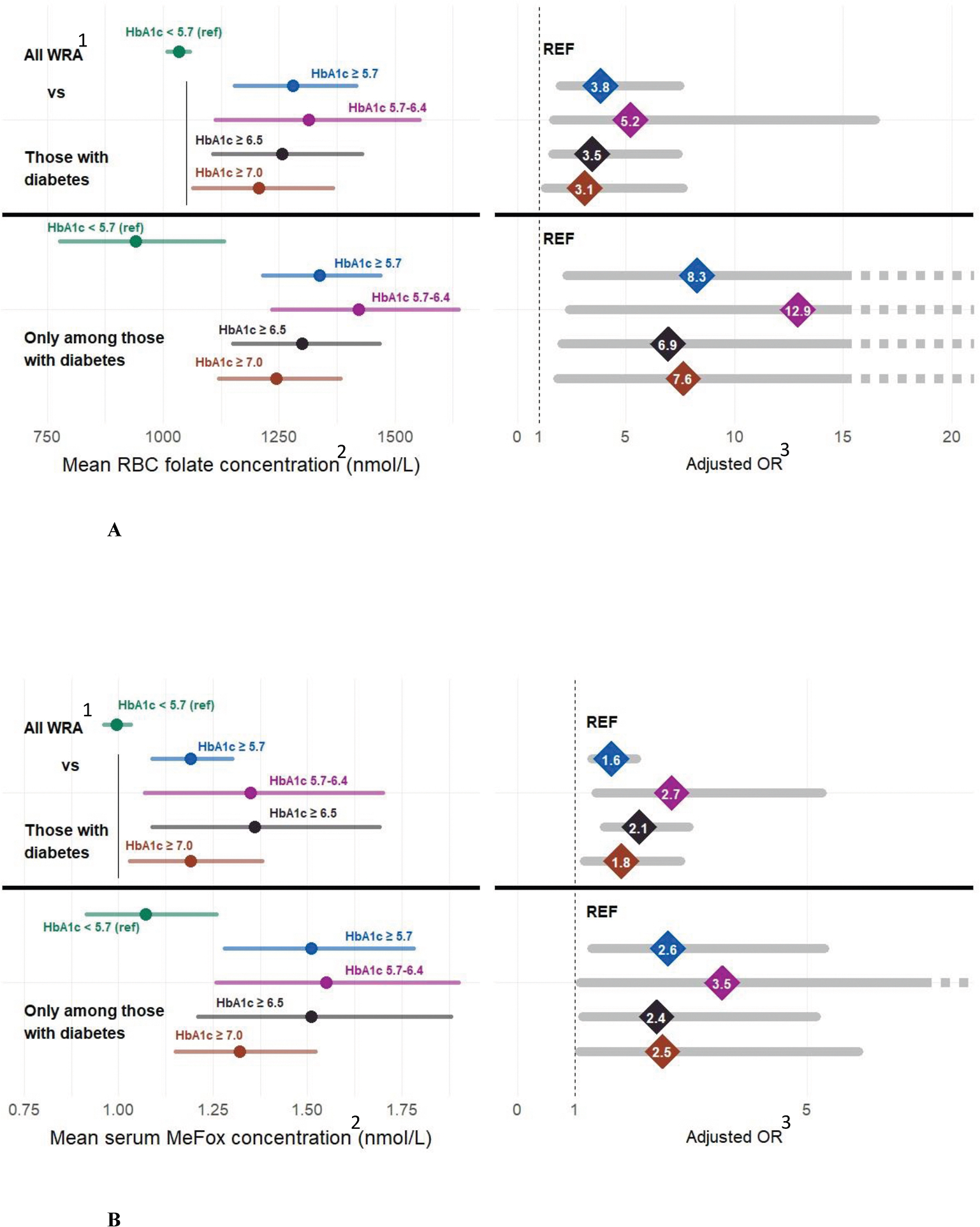
(A) Adjusted RBC folate concentrations (mean and 95% CI) among nonpregnant WRA, 12–49 y by HbA1c: NHANES 2011–March 2020. Euglycemia (<5.7) was associated with a reduction in >90th RBC folate concentration^2^ among those with diabetes [≥5.7: aOR of 8.3 (95% CI: 2.3, 29.9)], 5.7–6.4: aOR of 12.9 (95% CI: 2.4, 69.7), ≥6.5: aOR of 6.9 (95% CI: 2.1, 23.4), ≥7: aOR of 7.6 (95% CI: 1.9, 31.3)]. (B) Adjusted serum MeFox concentrations (mean and 95% CI) among nonpregnant WRA, 12–49 y by HbA1c: NHANES 2011–March 2020. Euglycemia (<5.7) was associated with reduction in >90th serum MeFox^2^ concentration among those with diabetes [≥5.7: aOR of 2.6 (95% CI: 1.3, 5.3), 5.7–6.4: aOR of 3.5 (95% CI: 1.1, 11.5), ≥6.5: aOR of 2.4 (95% CI: 1.1, 5.2), ≥7 aOR of 2.5 (95% CI: 1.1, 5.9)]. ^1^Those with controlled diabetes and those without diabetes (excludes participants with prediabetes). ^2^Adjusted for age, race, BMI, family-income-poverty ratio, smoking status, eGFR, and supplement use. ^3^Adjusted for age, race, BMI, eGFR, and supplement use. aOR, adjusted odds ratio; CI, confidence interval; eGFR, estimated glomerular filtration rate; HbA1c, glycated hemoglobin; MeFox, pyrazino-s-triazine derivative of 4-alpha-hydroxy-5-methyltetrahydrofolate; RBC, red blood cell; WRA, women of reproductive age.

When examining the association of diabetes control (at 3 different cut points for euglycemia HbA1c <5.7%, <6.5% and <7% mg/dL) on the RBC folate concentrations and serum MeFox of WRA with diabetes, higher HbA1c was associated with higher RBC folate and serum MeFox concentrations (Figure 3A, B and Supplemental Table 4). WRA with diabetes who had moderate control (HbA1c <6.5%) still had both elevated RBC folate and serum MeFox concentrations (Figure 3A, B). RBC folate concentrations were higher among WRA on multiple (≥5) diabetes medications (Supplemental Table 5).

## Discussion

Our analyses found that ~4.2 million (5.3%) nonpregnant WRA in the United States had diabetes, and among those ~1.4 million (1.8%) had undiagnosed diabetes. Overall, ~2.5 million (3.1%) WRA are estimated to have uncontrolled diabetes, and if they become pregnant, they are at high risk of adverse outcomes for themselves and their babies [9,50]. We found that ~25.8 million (32.3%) WRA were estimated to have prediabetes. Previous studies have indicated that over half of these could eventually progress to diabetes [51,52]. Among WRA with diabetes, ~3.0 million (70.9%) were not taking any supplements containing FA. Our analysis found evidence that WRA with uncontrolled diabetes may have altered folate metabolism with the higher concentrations of an oxidized form of folate (MeFox). The Institute of Medicine and the United States Preventive Service Task Force recommend that females who can become pregnant consume 400 μg/d FA as standard of care [53,54]. Because >40% of pregnancies in the United States are unintended, and the early closure of the neural tube in pregnancy, consumption of FA needs to begin before pregnancy and continue throughout the critical developmental time periods to prevent birth defects [9,50,55–58]. Given that diabetes and prediabetes are common in WRA, routine glucose screening during prepregnancy and early gestation could enable appropriate management [9,50,55–58].

### Implications for research

Biomarker surveillance of nutrients is complex and requires an understanding of intake, metabolism, and utilization, and risk may vary by condition. In addition, forms of folate other than FA are highly unstable to heat, light, and oxidation, in food, blood, and in supplements, complicating analysis and interpretation [19,20,59–61]. We examined differences of all folate forms available in NHANES by diabetes status, including summary folate measures such as RBC folate and serum folate concentrations [3,19,20,59,62]. We also examined individual serum forms [e.g., 5-methyltetrahydrofolate (main form in serum), intermediate nonmethyl folate forms (5-for-myltetrahydrofolate, tetrahydrofolate, 5,10-Methenyltetrahydrofolate), UMFA (in serum awaiting excretion by kidney or conversion to dihydrofolate) and serum MeFox (oxidized inactive 5-methyltetrahydrofolate)] [3,19,20,62,63]. RBC folate and serum MeFox concentrations were elevated among those with diabetes across demographics and did not vary by FA or usual total folate intake. This association was ameliorated among individuals with glycemic control. Serum folate and UMFA did not follow this pattern. Generally, higher blood folates are a result of higher folate intake, and clear dose responses have been described [64,65]. Previous observational studies have associated higher BMI with higher RBC folate concentrations [66], with some hypothesizing differing cellular folate uptake due to altered metabolic states [67,68]. Altered 1-carbon metabolisms have been seen with other clinical conditions [21,22,62,69–72] and can result in higher RBC folate concentrations than expected without an associated higher intake [21,22,62,69–72]. Although diabetes is well known to alter metabolic pathways, this is the first report we are aware of describing associations between diabetes and alterations in folate metabolism. These findings suggest that caution should be used when associating high RBC folate concentration with adverse clinical outcomes in observational studies due to a lack of causal inference and potential artifacts of metabolic or other clinical conditions [7,28–31]. Randomized controlled trials are needed to establish causality between folate intake, diabetic control, folate biomarker concentrations, and health outcomes.

### Implications for public health monitoring

RBC folate concentrations are critical for monitoring FA fortification programs and estimating NTD risk [24,59,60,64,73]. The OBF threshold has been established by the WHO as >906 nmol/L (>748 nmol/L with NHANES folate assay). Our study found that the overall impact of misclassification of OBF at the population level due to diabetes diagnosis is limited; excluding WRA with diabetes only increases the percent of those under the threshold to 17.98% (95% CI: 16.11%, 20.02%) from 17.63% (95% CI: 15.84%, 19.58%). The finding of higher folate concentrations among those with diabetes will have limited impact on WHO OBF monitoring.

### Implications for clinicians and individuals

Studies have found FA supplementation reduced the risk of birth defects associated with pregestational diabetes [5,6,74]. FA is also effective at preventing NTDs in populations with high rates of vitamin B_12_ deficiency [3,23,27,75]. The American Diabetes Association recommends that females with diabetes planning to become pregnant achieve as near normal glucose concentrations as possible, before and during pregnancy, to prevent birth defects and adverse pregnancy outcomes in addition to taking 400 μg/d FA [9]. Many countries recommend high-dose FA supplementation (5000 μg/d) for those with diabetes planning pregnancy; the current recommendation in the United States is 400 μg/d [76,77]. The United States limits recommendations of 4000 μg/d for females with a previous pregnancy with an NTD [78]. Additional research on the impact of different dosages of FA among those with pregestational diabetes is needed. This report supports these recommendations and highlights the number of females at risk for adverse outcomes. Our study found that almost half (47.7%) of WRA with diabetes had HbA1c concentration above the cut point of 7% mg/dL, indicating inadequate control of diabetes. When the cut point was set at <5.7% mg/dL (euglycemia), this percentage rose to 89.3%. Recent advancements in diabetes treatment, such as continuous glucose monitoring and insulin pumps, have not been widely studied during pregnancy [79,80]. Furthermore, research is needed to identify opportunities to achieve euglycemia before and during pregnancy to reduce the risk of birth defects, including the use of novel treatment methods. Research on the impact of diabetes medication during pregnancy is ongoing [81–83]. Pregestational diabetes (type 1 and type 2) poses maternal and fetal risks that are directly related to hyperglycemia [1,18,84–86]. Screening and prevention efforts are also critical in the overall population, given the high percentage of females with prediabetes at risk for progression to full diabetes before their next pregnancy.

### Strengths/limitations

NHANES is a large population-based ongoing survey of the civilian noninstitutionalized United States population. This provides thousands of data points on self-reported demographics such as age and multiple measured biomarkers and nutritional intakes, with sufficient sampling to examine somewhat rare outcomes such as diabetes in younger populations. However, even with a sample of 3731 WRA with complete data, some strata had a limited sample size that can produce unstable estimates, as was noted in multiple tables. Not all biomarkers of interest were present in every survey year (e.g., we excluded serum vitamin B_12_ since data were only available for 2011–2014). In addition, we were unable to fully identify WRA with diabetes by repeated glucose testing. Lastly, the study’s cross-sectional design renders it impossible to establish a causal inference from the results.

## Conclusions

Increasing maternal age and pregnancy BMI may be placing a higher proportion of females at risk of pregnancy complications due to diabetes. Diabetes was associated with high RBC folate concentration and low FA intake and high MeFox; however, this was ameliorated among those with good glycemic control. Screening for diabetes, preventing diabetes among those at risk, and ensuring tight glycemic control among those with diabetes, before and during pregnancy, could have high potential to mitigate adverse outcomes.

## Supplementary Material

SUP - Crider - Prediabetes, diabetes, and folate status among United States women of

## Figures and Tables

**TABLE 1:** Prevalence of diabetes and prediabetes by characteristics of nonpregnant women of reproductive age, 12–49 y: NHANES 2011-March 2020

Characteristics and risk factors	Overall (*n* = 3731)		Weighted % (95% CI)^1^		
			Diabetes^2^ (*n* = 229)	Prediabetes^2^ (*n* = 1210)	No diabetes^2^ (*n* = 2292)
			
	*n*	Weighted %	5.3 (4.4, 6.3)	32.3 (30.0, 34.7)	62.4 (59.9, 64.8)

Age (y)					
<35	2365	60.8	1.9 (1.3, 2.8)^3^	27.2 (24.5, 30.2)	70.9 (67.9, 73.7)
≥35	1366	39.2	10.5 (8.5, 12.8)	40.3 (37.1, 43.5)	49.3 (46.2, 52.3)
Age (y)					
<25	1504	34.6	0.8 (0.5, 1.4)^3, 4^	24.1 (20.9, 27.6)	75.1 (71.5, 78.3)
25–29	415	13.0	2.5 (1.2, 5.2)^3, 4^	27.9 (23.2, 33.2)	69.6 (64.1, 74.6)
30–34	446	13.3	4.3 (2.5, 7.2)^3, 4^	34.7 (28.9, 41.0)	61.0 (54.6, 67.0)
≥35	1366	39.2	10.5 (8.5, 12.8)	40.3 (37.1, 43.5)	49.3 (46.2, 52.3)
Race/ethnicity					
NH White	1157	55.7	4.0 (2.9, 5.5)^3^	30.0 (26.8, 33.5)	65.9 (62.5, 69.2)
NH Black	875	13.6	7.0 (5.3, 9.3)	34.8 (31.3, 38.5)	58.2 (54.1, 62.1)
NH Asian	506	6.3	4.2 (2.7, 6.4)^3, 4^	32.8 (27.5, 38.6)	63.0 (57.8, 67.9)
All Hispanic^5^	1013	20.1	7.9 (6.2, 10.0)	35.5 (31.8, 39.4)	56.6 (52.9, 60.2)
NH Other^5^	180	4.3	5.7 (2.7, 11.4)^3, 4, 6^	38.6 (28.8, 49.5)	55.7 (45.8, 65.1)
Family-income-poverty ratio	3425				
<1.0	1011	21.4	7.1 (5.5, 9.0)	34.3 (30.7, 38.1)	58.7 (54.7, 62.5)
1–1.9	860	21.8	5.4 (4.0, 7.5)^3^	31.6 (27.7, 35.9)	62.9 (58.6, 67.1)
2–3.9	859	28.1	5.5 (3.7, 8.1)^3^	35.4 (30.9, 40.1)	59.1 (54.3, 63.7)
≥4.0	695	28.7	3.6 (2.2, 5.7)^3^	27.1 (23.5, 31.1)	69.3 (65.3, 73.1)
Smoking exposure^7^	3690				
Nonsmoker	2866	76.9	5.0 (4.0, 6.1)	31.0 (28.5, 33.6)	64.0 (61.3, 66.7)
Smoker	824	23.1	6.1 (4.3, 8.7)	37.3 (32.5, 42.4)	56.6 (51.3, 61.7)
BMI^8^ (kg/m^2^)	3678				
Under/normal weight	1465	40.6	0.7 (0.3, 1.4)^3, 4^	20.5 (17.8, 23.5)	78.8 (75.9, 81.5)
Overweight	873	23.5	3.8 (2.6, 5.5)^3^	34.4 (30.3, 38.8)	61.8 (57.0, 66.3)
Obesity	1340	35.9	11.2 (9.2, 13.5)	44.8 (41.2, 48.4)	44.1 (40.4, 47.7)
Optimal RBC folate (nmol/L)					
≥748	2965	82.4	5.7 (4.7, 6.9)	32.0 (29.5, 34.6)	62.3 (59.7, 64.9)
<748	766	17.6	3.4 (2.3, 5.0)^3^	34.0 (29.0, 39.3)	62.6 (57.2, 67.7)
RBC folate (nmol/L)					
>90th percentile: >1700	290	10.3	14.8 (9.7, 21.7)^3^	35.4 (27.3, 44.4)	49.9 (42.0, 57.7)
≤90th percentile	3441	89.7	4.2 (3.5, 5.0)	32.0 (29.7, 34.3)	63.8 (61.4, 66.2)
RBC folate (nmol/L)					
>95th percentile: >1965	142	5.1	18.0 (10.6, 28.8)^3, 4^	36.3 (26.9, 46.8)	45.8 (35.2, 56.7)
≤95th percentile	3589	94.9	4.6 (3.9, 5.4)	32.1 (29.8, 34.5)	63.3 (60.8, 65.7)
Serum MeFox (nmol/L)					
>90th percentile: >2.2	325	10.0	12.8 (8.8, 18.2)^3^	35.4 (29.6, 41.7)	51.8 (44.5, 59.0)
≤90th percentile	3352	90.0	4.5 (3.7, 5.4)	32.1 (29.6, 34.7)	63.4 (60.8, 65.9)
Serum MeFox (nmol/L)					
>95th percentile: >2.9	156	5.0	12.4 (7.0, 21.0)^3, 4^	36.1 (27.1, 46.1)	51.5 (41.0, 61.8)
≤95th percentile	3251	95.0	4.9 (4.1, 6.0)	32.3 (29.8, 34.8)	62.8 (60.2, 65.3)
Serum B12^9^ (nmol/L)	1218				
Deficiency (<148)	21	2.1	12.3 (2.8, 40.3)^3,4, 6^	31.0 (11.7, 60.3)^3, 4, 6^	56.7 (34.3, 76.7)^3, 4^
Marginal/insufficiency (148–258)	218	19.5	4.3 (2.2, 8.6)^3, 4^	36.2 (28.7, 44.5)	59.4 (51.2, 67.2)
Sufficient (>258)	979	78.5	5.6 (4.1, 7.6)	30.5 (27.4, 33.9)	63.9 (59.7, 67.9)
Serum MMA^9^ (nmol/L)	1218				
Elevated MMA (>210)	89	7.5	9.4 (4.2, 19.4)^3, 4^	28.9 (17.2, 44.1)^3, 4^	61.8 (47.4, 74.3)
Nonelevated MMA (≤210)	1129	92.5	5.1 (3.9, 6.8)	31.9 (28.9, 35.0)	63.0 (59.1, 66.7)
eGFR^10^ (mL/min/1.73 m^2^)					
Normal (90 or higher)	3336	88.7	5.0 (4.1, 6.1)	32.1 (29.6, 34.6)	62.9 (60.2, 65.5)
Mild loss (60–89)	313	10.9	6.4 (3.9, 10.5)^3, 6^	36.6 (30.2, 43.4)	57.0 (49.5, 64.1)
Moderate loss-kidney failure (15–59)	20	0.4	22.0 (8.1, 47.7)^3, 4, 6^	25.8 (9.9, 52.6)^3, 4, 6^	52.1 (27.0, 76.2)^3, 4, 6^
ACR^11^ (mg/g)	3696				
<30	3300	90.8	4.5 (3.6, 5.7)	32.8 (30.4, 35.3)	62.7 (60.1, 65.3)
30–300	350	8.3	11.5 (8.1, 15.9)^3^	26.8 (21.5, 32.7)	61.8 (55.1, 68.1)
>300	46	0.9	28.9 (16.0, 46.5)^3, 4, 6^	26.2 (14.9, 41.8)^3, 4^	44.8 (30.7, 59.9)^3, 4^
Supplement use					
Yes	914	27.9	5.5 (4.0, 7.5)	28.5 (24.2, 33.3)	65.9 (61.3, 70.3)
No	2817	72.1	5.2 (4.3, 6.3)	33.8 (31.2, 36.5)	61.0 (58.0, 63.9)
Supplement use (μg/d)					
≥400	400	12.3	8.1 (5.3, 12.4)^3^	30.7 (25.6, 36.3)	61.2 (55.5, 66.6)
<400	3331	87.7	4.9 (4.1, 5.9)	32.6 (30.2, 35.0)	62.6 (59.9, 65.2)
Supplement use (μg/d)					
<400	514	15.5	3.4 (2.1, 5.6)^3, 4^	26.8 (21.4, 33.1)	69.7 (63.0, 75.6)
≥400	400	12.3	8.1 (5.3, 12.4)^3^	30.7 (25.6, 36.3)	61.2 (55.5, 66.6)
Nonuser	2817	72.1	5.2 (4.3, 6.3)	33.8 (31.2, 36.5)	61.0 (58.0, 63.9)
Folic acid consumption group^12^	3473				
ECGP/CMF	1914	53.5	5.8 (4.7, 7.0)	34.9 (31.2, 38.8)	59.3 (55.4, 63.1)
ECGP/CMF + RTE	710	18.5	3.6 (2.4, 5.6)^3^	29.8 (26.2, 33.8)	66.5 (62.3, 70.5)
ECGP/CMF + Sup	650	21.0	5.4 (3.7, 7.8)^3^	29.0 (24.1, 34.5)	65.6 (59.9, 70.8)
ECGP/CMF + RTE + Sup	199	6.9	5.6 (2.7, 11.4)^3, 4, 6^	25.2 (18.5, 33.5)^3^	69.2 (60.7, 76.5)
RBC folate-folate intake					
>90th percentile and ≥400 μg	114	3.9	14.9 (8.0, 26.0)^3, 4^	28.8 (18.6, 41.7)^3^	56.3 (44.1, 67.8)
>90th percentile and <400 μg	176	6.5	14.7 (8.9, 23.3)^3, 4^	39.3 (30.7, 48.6)	46.0 (37.0, 55.3)
≤90th percentile and ≥400 μg	286	8.5	5.1 (3.0, 8.5)^3, 4, 6^	31.5 (26.4, 37.1)	63.4 (57.6, 68.9)
≤90th percentile and <400 μg	3155	81.2	4.1 (3.5, 4.9)	32.0 (29.7, 34.5)	63.9 (61.3, 66.3)
Geometric mean (95% CI)^1^					
	*n*	Overall	Diabetes^2^	Prediabetes^2^	No diabetes^2^

RBC folate (nmol/L)	3731	1043 (1019, 1066)	1271 (1164, 1378)	1036 (1002, 1071)	1028 (1000, 1056)
Serum folate (nmol/L)	3674	36 (35, 37)	35 (31, 40)	34 (32, 35)	37 (36, 38)
RBC-to-serum folate ratio^13^	3674	29 (29, 30)	36 (32, 40)	31 (30, 32)	28 (27, 28)
Serum MeFox (nmol/L)	3677	1.02 (0.98, 1.06)	1.43 (1.23, 1.63)	1.03 (0.98, 1.09)	0.98 (0.94, 1.03)
UMFA (nmol/L)	3674	0.66 (0.64, 0.68)	0.65 (0.57, 0.73)	0.64 (0.61, 0.68)	0.66 (0.64, 0.69)
5-Methyltetrahydrofolate (nmol/L)	3677	34 (33, 35)	33 (29, 37)	31 (30, 33)	35 (34, 36)
5-Formyltetrahydrofolate (nmol/L)	3677	0.16 (0.15, 0.16)	0.16 (0.15, 0.16)	0.16 (0.15, 0.16)	0.16 (0.15, 0.16)
Tetrahydrofolate (nmol/L)	3677	0.67 (0.63, 0.72)	0.80 (0.69, 0.92)	0.68 (0.63, 0.73)	0.66 (0.61, 0.71)
5,10-Methenyltetrahydrofolate (nmol/L)	3677	0.18 (0.17, 0.18)	0.18 (0.17, 0.19)	0.18 (0.17, 0.18)	0.18 (0.17, 0.18)

Abbreviations: ACR, albumin-to-creatinine ratio; CI, confidence interval; CMF, corn masa flour; ECGP, enriched cereal-grain products; eGFR, estimated glomerular filtration rate; HbA1c, glycated hemoglobin; MeFox, pyrazino-s-triazine derivative of 4-alpha-hydroxy-5-methyltetrahydrofolate; MMA, methylmalonic acid; NH, non-Hispanic; RBC, red blood cell; RTE, ready-to-eat; SUP, folic acid-containing supplements; UMFA, unmetabolized folic acid.

1 Unadjusted percentages and geometric means are weighted, and CIs accounting for complex sampling design.

2 Categorized using the Diabetes NHANES questionnaire, fasting blood sugar, and glycohemoglobin concentration (HbA1c); includes self-reported (*n* = 151) and undiagnosed (*n* = 78).

3 Degree of freedom <8.

4 Sample size <30.

5 “Hispanic” includes respondents who self-identified as “Mexican American” and self-identified “Hispanic” ethnicity. NH participants were categorized based on their self-reported races: “NH Other” (including multiple races).

6 Does not meet the criteria for prevalence estimate reliability.

7 Smoking exposure: Biomonitoring Summary | CDC, cotinine concentrations- (nonsmoker ≤10 ng/mL) and smoker ≥10 ng/mL).

8 BMI categories for NH Asian participants were based on the WHO expert consultation: Appropriate body-mass index for Asian populations and its implications for policy and intervention strategies - PubMed (nih.gov)

9 NHANES dataset available for 2011–2012.

10 eGFR based on the Chronic Kidney Disease Epidemiology Collaboration (CKD-EPI) creatitine equation (2021).

11 ACR based on the National Kidney Foundation—https://www.kidney.org/kidneydisease/siemens_hcp_acr.

12 ECGP/CMF only, consumed enriched cereal-grain products/corn masa flour only, excluding ready-to-eat cereals and supplements containing folic acid; ECGP/CMF+RTE, consumed enriched cereal-grain products/corn masa flour plus ready-to-eat cereals; ECGP/CMF+SUP, consumed enriched cereal-grain products/corn masa flour (excluding ready-to-eat cereals) plus supplements containing folic acid; ECGP/CMF+RTE+SUP, consumed enriched cereal-grain products/corn masa flour, ready-to-eat cereals, and supplements containing folic acid.

13 RBC-to-serum folate ratio: adjusts for intrapersonal variations.

**TABLE 2:** aOR for diabetes compared with no diabetes, nonpregnant women of reproductive age, 12–49 y: NHANES 2011-March 2020

	aOR (95% CI)	*P* value

RBC folate-folate intake^1^		
>90th percentile and ≥400 μg	2.19 (0.93, 5.18)	0.0732
>90th percentile and <400 μg	2.28 (1.23, 4.24)	0.0097
≤90th percentile and ≥400 μg	1.11 (0.59, 2.07)	0.7434
≤90th percentile and <400 μg	Reference	—
Race/ethnicity		
NH Black	2.15 (1.26, 3.67)	0.0054
NH Asian	2.07 (1.12, 3.82)	0.0214
Hispanic^2^	2.54 (1.64, 3.92)	0.0001
NH White	Reference	—
Age^3^	1.12 (1.08, 1.15)	<0.0001
BMI (kg/m^2^)	1.08 (1.07, 1.10)	<0.0001
eGFR^4^	1.02 (1.00, 1.04)	0.0574
Serum MeFox^5^ (nmol/L)	1.84 (1.38, 2.45)	0.0001
Family-income-poverty ratio	0.89 (0.77, 1.02)	0.0812

Sample size, *n* = 3296.

Abbreviations: aOR, adjusted odds ratio; CI, confidence interval; eGFR, estimated glomerular filtration rate; MeFox, pyrazino-s-triazine derivative of 4-alpha-hydroxy-5-methyltetrahydrofolate; NH, non-Hispanic; RBC, red blood cell.

1 >90th percentile: >1700 nmol/L.

2 “Hispanic” includes respondents self-identified as “Mexican American” and self-identified “Hispanic” ethnicity. NH participants were categorized based on their self-reported races.

3 Age in years.

4 eGFR based on the Chronic Kidney Disease Epidemiology Collaboration (CKD-EPI) creatitine equation (2021).

5 Log transformed.

**TABLE 3:** aOR for red blood cell folate >90th compared with ≤90th percentile, nonpregnant women of reproductive age, 12–49 y: NHANES 2011-March 2020

	aOR (95% CI)	*P* value

Diabetes status		
Diabetes	2.64 (1.46, 4.77)	0.0017
No diabetes	Reference	—
Race/ethnicity		
NH Black	0.23 (0.14, 0.36)	<0.0001
NH Asian	0.40 (0.25, 0.66)	0.0004
Hispanic^1^	0.43 (0.29, 0.65)	0.0001
NH White	Reference	—
Age^2^	1.04 (1.02, 1.06)	0.0001
BMI (kg/m^2^)	1.02 (1.00, 1.04)	0.0145
Folic acid (μg/d)		
≥400	5.01 (3.36, 7.47)	<0.0001
< 400	Reference	—

Sample size, *n* = 3678.

Abbreviations: aOR, adjusted odds ratio; CI, confidence interval; NH, non-Hispanic.

1 “Hispanic” includes respondents self-identified as “Mexican American” and self-identified “Hispanic” ethnicity. NH participants were categorized based on their self-reported races.

2 Age in years.

**TABLE 4:** aOR for serum MeFox >90th compared with ≤90th percentile, nonpregnant women of reproductive age, 12–49 y: NHANES 2011-March 2020

	aOR (95% CI)	*P* value

Diabetes status		
Diabetes	2.09 (1.33, 3.28)	0.0018
No diabetes	Reference	—
Race/ethnicity		
NH Black	0.44 (0.31, 0.62)	<0.0001
NH Asian	0.65 (0.40, 1.05)	0.0758
Hispanic^1^	0.38 (0.26, 0.55)	<0.0001
NH White	Reference	—
Age^2^	1.02 (1.00, 1.03)	0.0948
BMI (kg/m^2^)	1.05 (1.03, 1.06)	<0.0001
Folic acid (μg/d)		
≥400	1.35 (0.92, 1.98)	0.1278
<400	Reference	—

Sample size, *n* = 3626.

Abbreviations: aOR, adjusted odds ratio; CI, confidence interval; MeFox, pyrazino-s-triazine derivative of 4-alpha-hydroxy-5-methyltetrahydrofolate; NH, non-Hispanic.

1 “Hispanic” includes respondents self-identified as “Mexican American” and self-identified “Hispanic” ethnicity. NH participants were categorized based on their self-reported races.

2 Age in years.

## Data Availability

Publicly available datasets were analyzed in this study. The data can be found here: https://wwwn.cdc.gov/nchs/nhanes/default.aspx (accessed 22 November, 2022). Institute review board approvals NHANES—National Center for Health Statistics Research Ethics Review Board Approval (cdc.gov).

## Appendix A. Supplementary data

Supplementary data to this article can be found online at https://doi.org/10.1016/j.ajcnut.2026.101193.

## Abbreviations

aOR: adjusted odds ratio
CI: confidence interval
CMF: corn masa flour
DFE: dietary folate equivalents
DNS: data not shown
ECGP: enriched cereal-grain products
eGFR: estimated glomerular filtration rate
FA: folic acid
FIPR: family-income-poverty ratio
FPG: fasting plasma glucose
HbA1c: glycated hemoglobin
MeFox: pyrazino-*s*-triazine derivative of 4-alpha-hydroxy-5-methyltetrahydrofolate
NCI: National Cancer Institute
NH: non-Hispanic
NTD: neural tube defect
RBC: red blood cell
RTE: ready-to-eat cereal
SUP: folic acid-containing supplements
UMFA: unmetabolized folic acid
WRA: women of reproductive age
