# Supplementary material for: Prediabetes, diabetes, and folate status among United States women of reproductive age: NHANES 2011–March 2020
Source: Am J Clin Nutr. Author manuscript; Available in PMC 2026 Mar 30. (PMC13034014; doi:10.1016/j.ajcnut.2026.101193)
Supplement: SUP - Crider - Prediabetes, diabetes, and folate status among United States women of [file NIHMS2154132-supplement-SUP_-_Crider_-_Prediabetes__diabetes__and_folate_status_among_United_States_women_of.docx]

**Supplemental Table 1**

**Prevalence of diabetes and prediabetes by characteristics of non-pregnant women of reproductive Age, 12–49:**

**National Health and Nutrition Examination Survey 2011–March 2020**

| Characteristics and risk factors | Overall  (n = 3731) | | Diabetes^1^  (n = 229) | | Prediabetes^1^  (n = 1210) | | No Diabetes^1^  (n = 2292) | |
| --- | --- | --- | --- | --- | --- | --- | --- | --- |
|  | n | Weighted % (95% CI)^2^ | n | Weighted % (95% CI)^2^ | n | Weighted % (95% CI)^2^ | n | Weighted % (95% CI)^2^ |
| Age (years) |  |  |  |  |  |  |  |  |
| <35 | 2365 | 60.8 (58.8-62.7) | 51 | 22.2 (15.8-30.2)^3^ | 657 | 51.2 (47.5-54.9) | 1657 | 69.1 (66.9-71.2) |
| ≥35 | 1366 | 39.2 (37.3-41.2) | 178 | 77.8 (69.8-84.2) | 553 | 48.8 (45.1-52.5) | 635 | 30.9 (28.8-33.1) |
| Age (years) |  |  |  |  |  |  |  |  |
| <25 | 1504 | 34.6 (32.3-37.0) | 16 | 5.4 (3.1-9.1)^4^ | 373 | 25.8 (22.8-29.0) | 1115 | 41.6 (38.7-44.6) |
| 25-29 | 415 | 13.0 (11.4-14.7) | 12 | 6.1 (3.0-12.0)^4^ | 123 | 11.2 (8.9-14.0) | 280 | 14.5 (12.5-16.7) |
| 30-34 | 446 | 13.3 (11.8-14.9) | 23 | 10.7 (6.4-17.4)^4^ | 161 | 14.2 (11.5-17.5) | 262 | 13.0 (11.4-14.8) |
| ≥35 | 1366 | 39.2 (37.3-41.2) | 178 | 77.8 (69.8-84.2) | 553 | 48.8 (45.1-52.5) | 635 | 30.9 (28.8-33.1) |
| Race/Ethnicity | 3731 |  | 229 |  | 1210 |  | 2292 |  |
| NH White | 1157 | 55.7 (51.6-59.7) | 54 | 42.2 (32.9-52.1)^3^ | 341 | 51.8 (46.4-57.0) | 762 | 58.9 (54.8-62.8) |
| NH Black | 875 | 13.6 (11.4-16.2) | 69 | 18.1 (13.2-24.4) | 300 | 14.7 (11.8-18.1) | 506 | 12.7 (10.5-15.3) |
| NH Asian | 506 | 6.3 (5.2-7.6) | 20 | 5.0 (2.9-8.3)^4^ | 165 | 6.4 (5.0-8.2) | 321 | 6.4 (5.2-7.9) |
| All Hispanic^5^ | 1013 | 20.1 (17.2-23.3) | 76 | 30.1 (23.3-37.9) | 347 | 22.0 (18.3-26.3) | 590 | 18.2 (15.5-21.3) |
| NH Other^5^ | 180 | 4.3 (3.4-5.3) | 10 | 4.6 (2.3-9.0)^4, 6^ | 57 | 5.1 (3.5-7.5) | 113 | 3.8 (3.0-4.9) |
| Family-income-poverty ratio | 3425 |  | 210 |  | 1093 |  | 2122 |  |
| <1.0 | 1011 | 21.4 (19.3-23.7) | 76 | 28.7 (21.5-37.1) | 346 | 23.0 (20.0-26.2) | 589 | 20.0 (17.8-22.5) |
| 1-1.9 | 860 | 21.8 (19.7-24.0) | 55 | 22.5 (15.9-30.9)^3^ | 276 | 21.6 (18.3-25.3) | 529 | 21.9 (19.4-24.5) |
| 2-3.9 | 859 | 28.1 (25.4-30.9) | 48 | 29.4 (21.3-39.0)^3^ | 286 | 31.1 (26.7-35.8) | 525 | 26.5 (23.7-29.4) |
| ≥4.0 | 695 | 28.7 (25.7-31.9) | 31 | 19.4 (13.1-27.9)^3^ | 185 | 24.3 (20.5-28.7) | 479 | 31.7 (28.1-35.4) |
| Smoking exposure^7^ | 3690 |  | 225 |  | 1196 |  | 2269 |  |
| Non-smoker | 2866 | 76.9 (74.6-79.0) | 169 | 73.0 (64.0-80.5) | 910 | 73.4 (69.2-77.2) | 1787 | 79.0 (76.3-81.5) |
| Smoker | 824 | 23.1 (21.0-25.4) | 56 | 27.0 (19.5-36.0) | 286 | 26.6 (22.8-30.8) | 482 | 21.0 (18.5-23.7) |
| BMI^8^ (kg/m^2^) | 3678 |  | 221 |  | 1203 |  | 2254 |  |
| Under/Normal weight | 1465 | 40.6 (38.4-42.9) | 12 | 5.2 (2.5-10.4)^4^ | 308 | 25.6 (22.8-28.7) | 1145 | 51.4 (48.5-54.2) |
| Overweight | 873 | 23.5 (21.8-25.3) | 41 | 17.2 (12.0-24.1)^3^ | 293 | 24.9 (22.1-27.9) | 539 | 23.3 (21.1-25.6) |
| Obesity | 1340 | 35.9 (33.9-37.8) | 168 | 77.6 (70.0-83.7) | 602 | 49.4 (46.0-52.9) | 570 | 25.3 (23.0-27.9) |
| Optimal RBC folate (nmol/L) | 3731 |  | 229 |  | 1210 |  | 2292 |  |
| ≥748 | 2965 | 82.4 (80.4-84.2) | 194 | 88.7 (83.4-92.5) | 968 | 81.5 (78.3-84.2) | 1803 | 82.3 (79.7-84.6) |
| <748 | 766 | 17.6 (15.8-19.6) | 35 | 11.3 (7.5-16.6)^3^ | 242 | 18.5 (15.8-21.7) | 489 | 17.7 (15.4-20.3) |
| RBC folate (nmol/L) | 3731 |  | 229 |  | 1210 |  | 2292 |  |
| >90th percentile: >1700 | 290 | 10.3 (8.8-12.1) | 45 | 28.9 (21.2-38.0)^3^ | 100 | 11.3 (8.7-14.6) | 145 | 8.3 (6.5-10.5) |
| ≤90th percentile | 3441 | 89.7 (87.9-91.2) | 184 | 71.1 (62.0-78.8) | 1110 | 88.7 (85.4-91.3) | 2147 | 91.7 (89.5-93.5) |
| RBC folate (nmol/L) | 3731 |  | 229 |  | 1210 |  | 2292 |  |
| >95th percentile: >1965 | 142 | 5.1 (4.1-6.2) | 25 | 17.3 (11.1-26.0)^4^ | 52 | 5.7 (4.2-7.7) | 65 | 3.7 (2.6-5.3) |
| ≤95th percentile | 3589 | 94.9 (93.8-95.9) | 204 | 82.7 (74.0-88.9) | 1158 | 94.3 (92.3-95.8) | 2227 | 96.3 (94.7-97.4) |
| Serum MeFox (nmol/L) | 3677 |  | 226 |  | 1193 |  | 2258 |  |
| >90th percentile: >2.2 | 325 | 10.0 (8.5-11.7) | 44 | 24.0 (17.4-32.3)^3^ | 117 | 10.9 (8.9-13.2) | 164 | 8.3 (6.5-10.5) |
| ≤90th percentile | 3352 | 90.0 (88.3-91.5) | 182 | 76.0 (67.7-82.6) | 1076 | 89.1 (86.8-91.1) | 2094 | 91.7 (89.5-93.5) |
| Serum MeFox (nmol/L) | 3677 |  | 226 |  | 1193 |  | 2258 |  |
| >95th percentile: >2.9 | 156 | 5.0 (4.0-6.3) | 20 | 11.7 (6.6-19.8)^4^ | 56 | 5.6 (4.0-7.8) | 80 | 4.2 (3.0-5.6) |
| ≤95th percentile | 3521 | 95.0 (93.7-96.0) | 206 | 88.3 (80.2-93.4) | 1137 | 94.4 (92.2-96.0) | 2178 | 95.8 (94.4-97.0) |
| Serum B_12_^9^ (nmol/L) | 1218 |  | 82 |  | 402 |  | 734 |  |
| Deficiency (<148) | 21 | 2.1 (1.1-3.8) | 2 | 4.7 (0.9-22.0)^4, 6^ | 6 | 2.0 (0.8-5.3)^4, 6^ | 13 | 1.9 (1.0-3.6)^4^ |
| Marginal/Insufficiency (148-258) | 218 | 19.5 (16.5-22.8) | 13 | 15.5 (7.2-30.2)^4^ | 79 | 22.3 (17.4-28.0) | 126 | 18.4 (14.7-22.8) |
| Sufficient (>258) | 979 | 78.5 (75.0-81.6) | 67 | 79.8 (70.3-86.9) | 317 | 75.7 (69.7-80.8) | 595 | 79.7 (75.3-83.5) |
| Serum MMA^9^ (nmol/L) | 1218 |  | 82 |  | 402 |  | 734 |  |
| Elevated MMA (>210) | 89 | 7.5 (5.5-10.2) | 11 | 12.9 (5.9-26.0)^4^ | 27 | 6.9 (3.7-12.4)^4^ | 51 | 7.4 (5.3-10.3) |
| Non-elevated MMA (≤210) | 1129 | 92.5 (89.8-94.5) | 71 | 87.1 (74.0-94.1) | 375 | 93.1 (87.6-96.3) | 683 | 92.6 (89.7-94.7) |
| eGFR^10^ (mL/min/1.73 m2) | 3669 |  | 224 |  | 1193 |  | 2252 |  |
| Normal (90 or higher) | 3336 | 88.7 (87.0-90.2) | 186 | 84.9 (77.5-90.2) | 1083 | 87.4 (84.3-90.1) | 2067 | 89.7 (87.4-91.6) |
| Mild loss (60-89) | 313 | 10.9 (9.4-12.6) | 32 | 13.3 (8.6-20.1)^3, 6^ | 105 | 12.2 (9.6-15.4) | 176 | 10.0 (8.1-12.2) |
| Moderate loss-Kidney failure (15-59) | 20 | 0.4 (0.3-0.7) | 6 | 1.8 (0.7-4.7)^4, 6^ | 5 | 0.3 (0.1-0.9)^4, 6^ | 9 | 0.4 (0.2-0.7)^4, 6^ |
| Albumin-creatinine ratio^11^ (mg/g) | 3696 |  | 226 |  | 1196 |  | 2274 |  |
| <30 | 3300 | 90.8 (89.6-91.9) | 170 | 77.3 (68.4-84.3) | 1079 | 92.4 (90.5-93.9) | 2051 | 91.2 (89.7-92.4) |
| 30-300 | 350 | 8.3 (7.3-9.4) | 43 | 18.0 (12.1-26.0) | 105 | 6.9 (5.5-8.7) | 202 | 8.2 (7.0-9.7) |
| >300 | 46 | 0.9 (0.6-1.2) | 13 | 4.7 (2.4-8.7)^4, 6^ | 12 | 0.7 (0.4-1.2)^4^ | 21 | 0.6 (0.4-1.0)^4^ |
| Supplement use | 3731 |  | 229 |  | 1210 |  | 2292 |  |
| Yes | 914 | 27.9 (25.8-30.1) | 60 | 29.1 (22.9-36.2) | 268 | 24.6 (20.8-28.9) | 586 | 29.5 (26.9-32.2) |
| No | 2817 | 72.1 (69.9-74.2) | 169 | 70.9 (63.8-77.1) | 942 | 75.4 (71.1-79.2) | 1706 | 70.5 (67.8-73.1) |
| Supplement use (μg/day) | 3731 |  | 229 |  | 1210 |  | 2292 |  |
| ≥400 | 400 | 12.3 (10.8-14.1) | 36 | 19.0 (13.0-27.0)^3^ | 128 | 11.7 (9.7-14.1) | 236 | 12.1 (10.2-14.3) |
| <400 | 3331 | 87.7 (85.9-89.2) | 193 | 81.0 (73.0-87.0) | 1082 | 88.3 (85.9-90.3) | 2056 | 87.9 (85.7-89.8) |
| Supplement use (μg/day) | 3731 |  | 229 |  | 1210 |  | 2292 |  |
| <400 | 514 | 15.5 (13.9-17.4) | 24 | 10.1 (6.2-16.0)^4^ | 140 | 12.9 (10.0-16.5) | 350 | 17.4 (15.3-19.6) |
| ≥400 | 400 | 12.3 (10.8-14.1) | 36 | 19.0 (13.0-27.0)^3^ | 128 | 11.7 (9.7-14.1) | 236 | 12.1 (10.2-14.3) |
| Non-user | 2817 | 72.1 (69.9-74.2) | 169 | 70.9 (63.8-77.1) | 942 | 75.4 (71.1-79.2) | 1706 | 70.5 (67.8-73.1) |
| Folic acid consumption group^12^ | 3473 |  | 213 |  | 1120 |  | 2140 |  |
| ECGP/CMF | 1914 | 53.5 (51.5-55.6) | 127 | 58.4 (50.7-65.7) | 658 | 58.3 (53.8-62.6) | 1129 | 50.7 (48.1-53.2) |
| ECGP/CMF+RTE | 710 | 18.5 (16.8-20.3) | 30 | 12.8 (8.8-18.2)^3^ | 219 | 17.2 (14.6-20.2) | 461 | 19.6 (17.4-22.1) |
| ECGP/CMF+Sup | 650 | 21.0 (19.2-22.9) | 44 | 21.5 (15.5-29.0)^3^ | 187 | 19.0 (15.6-22.9) | 419 | 22.0 (19.7-24.5) |
| ECGP/CMF+RTE+Sup | 199 | 6.9 (5.9-8.2) | 12 | 7.3 (3.7-14.0)^4, 6^ | 56 | 5.5 (3.9-7.7)^3^ | 131 | 7.7 (6.3-9.3) |
| RBC folate-folate intake | 3731 |  | 229 |  | 1210 |  | 2292 |  |
| >90th centile and ≥400 μg | 114 | 3.9 (3.0-5.0) | 16 | 10.9 (5.9-19.1)^4^ | 36 | 3.4 (2.2-5.3)^3^ | 62 | 3.5 (2.4-5.0) |
| >90th centile and <400 μg | 176 | 6.5 (5.2-8.0) | 29 | 18.0 (11.6-26.8)^4^ | 64 | 7.9 (5.8-10.5) | 83 | 4.8 (3.5-6.4) |
| ≤90th centile and ≥400 μg | 286 | 8.5 (7.3-9.8) | 20 | 8.1 (4.9-13.3)^4, 6^ | 92 | 8.3 (6.7-10.1) | 174 | 8.6 (7.2-10.3) |
| ≤90th centile and <400 μg | 3155 | 81.2 (78.9-83.3) | 164 | 63.0 (55.1-70.3) | 1018 | 80.4 (77.2-83.3) | 1973 | 83.1 (80.2-85.7) |

^1^Categorized using Diabetes NHANES questionnaire- fasting blood sugar and glycohemoglobin level (HbA1c); includes self-reported (n= 151) and undiagnosed (n = 78)

^2^ Unadjusted percentages and geometric means are weighted, and CIs accounting for complex sampling design.

^3^ Degree of freedom <8

^4^ Degree of freedom <8 and Sample size <30

^5^ “Hispanic” include respondents self-identified as “Mexican American” and self-identified “Hispanic” ethnicity. non-Hispanic (NH) participants were then categorized based on their self-reported races; “NH Other” (including multiple races).

^6^ Does not meet the criteria for prevalence estimate reliability

^7^ Smoking exposure: [Biomonitoring Summary | CDC](https://www.cdc.gov/environmental-exposure-report/index.html), Cotinine levels- (non-smoker = <10ng/ml) and smoker = >10ng/ml)

^8^ BMI categories for non-Hispanic Asian participants was based the WHO expert consultation: [Appropriate body-mass index for Asian populations and its implications for policy and intervention strategies - PubMed (nih.gov)](https://pubmed.ncbi.nlm.nih.gov/14726171/)

^9^ NHANES dataset available for 2011–2012

^10^ eGFR based on the National Kidney Foundation- CKD-EPI Creatinine Equation (2021)

^11^ ACR based on the National Kidney Foundation- <https://www.kidney.org/kidneydisease/siemens_hcp_acr>

^12^ ECGP/CMF only, consumed enriched cereal-grain products/corn masa flour only, excluding ready-to-eat cereals and supplements containing folic acid; ECGP/CMF+RTE, consumed enriched cereal-grain products/corn masa flour plus ready-to-eat cereals; ECGP/CMF+SUP, consumed enriched cereal-grain products/corn masa flour (excluding ready-to-eat cereals) plus supplements containing folic acid; ECGP/CMF+RTE+SUP, consumed enriched cereal-grain products/corn masa flour, ready-to-eat cereals, and supplements containing folic acid.

Abbreviations: Body mass index (BMI), Estimated glomerular filtration rate (eGFR), Methylmalonic acid (MMA), Pyrazino-s-triazine derivative of 4-alpha-hydroxy-5-methyltetrahydrofolate (MeFox), Red blood cell (RBC), Unmetabolized folic acid (UMFA)

**Supplemental Table 2**

**Adjusted Median Usual intake Among Non-pregnant Women of Reproductive Age, 12–49 Years: National Health and Nutrition Examination Survey 2011–March 2020**

|  | n | Median estimate (IQR)^1^ | | | |
| --- | --- | --- | --- | --- | --- |
|  |  | Total food folate^2^ (DFE/day) | Total folic acid^3^  (μg/day) | Total folate^4^  (μg/day) | Total folate^5^  (DFE/day) |
| Overall^6^ | 3473 | 173 (126-223) | 196 (92-343) | 390 (233-570) | 533 (300-814) |
| Age^7^ (years) |  |  | | | |
| <35 years | 2238 | 168 (124-216) | 195 (97-329) | 381 (237-546) | 523 (309-779) |
| ≥ 35 years | 1235 | 180 (132-231) | 198 (83-364) | 404 (231-604) | 548 (290-863) |
| Race/Ethnicity^8^ |  |  | | | |
| NH White | 1106 | 174 (128-225) | 211 (100-370) | 406 (245-599) | 559 (318-861) |
| NH Black | 822 | 152 (112-193) | 174 (83-295) | 340 (206-489) | 465 (266-697) |
| NH Asian | 433 | 192 (148-236) | 193 (88-329) | 406 (255-570) | 546 (321-802) |
| All Hispanic | 937 | 183 (129-239) | 178 (84-301) | 382 (234-539) | 512 (296-752) |
| NH Other | 175 | 153 (120-187) | 197 (93-345) | 370 (226-540) | 511 (293-785) |
| Folic acid consumption group^9^ | 3473 |  | | | |
| ECGP/CMF | 1914 | 162 (119-207) | 110 (79-144) | 275 (215-336) | 355 (278-432) |
| ECGP/CMF+RTE | 710 | 161 (112-214) | 249 (235-261) | 412 (348-474) | 594 (543-639) |
| ECGP/CMF+Sup | 650 | 196 (154-240) | 416 (242-629) | 631 (429-855) | 925 (599-1297) |
| ECGP/CMF+RTE+Sup | 199 | 202 (149-271) | 615 (438-822) | 839 (628-1079) | 1271 (940-1649) |
| BMI^10^ (kg/m^2^) | 3432 |  | | | |
| Under/Normal weight | 1366 | 180 (130-236) | 215 (105-369) | 418 (255-607) | 575 (332-867) |
| Overweight | 807 | 178 (134-225) | 188 (91-323) | 385 (242-550) | 522 (309-779) |
| Obesity | 1259 | 162 (120-206) | 186 (87-330) | 367 (218-543) | 502 (281-779) |
| Diabetes status^11^ |  |  | | | |
| Diabetes | 213 | 171 (126-220) | 197 (78-384) | 391 (212-616) | 536 (268-891) |
| Pre-diabetes | 1120 | 167 (124-211) | 179 (86-304) | 362 (227-512) | 490 (288-725) |
| No diabetes | 2140 | 175 (127-228) | 205 (96-359) | 402 (240-593) | 553 (312-847) |
|  |  |  |  |  |  |

^1^ Median intake and IQR accounting for the complex sampling design, adjustment for variations in day 1, day 2 measurements and by the day of the week; statistical analyses performed using Monte Carlo simulated pseudo-persons (each participant generated 100 pseudo-persons of the participant)

^2^Total food folate (DFE): Total natural folate from foods

^3^ Total folic acid (μg): Total folic acid from fortified foods and supplements (30d)

^4^ Total folate (μg): Total folate from foods (folic acid and natural food folate) and folic acid from supplements (30d)

^5^ Total folate (DFE): Total folate in DFEs from foods (folic acid and natural food folate) and folic acid from supplements (30d)

μg DFE = μg naturally occurring folate + (1.7 × μg folic acid from foods and supplements)

^6^Adjusted for adjusted for age, race, FIPR and BMI

^7^Adjusted for race, FIPR, BMI

^8^ Adjusted for age, FIPR and BMI; “Hispanic” include respondents self-identified as “Mexican American” and self-identified “Hispanic” ethnicity. non-Hispanic (NH) participants were then categorized based on their self-reported races; “NH Other” (including multiple races).

^9^ Adjusted for age, race, FIPR and BMI; ECGP/CMF only, consumed enriched cereal-grain products/corn masa flour only, excluding ready-to-eat cereals and supplements containing folic acid; ECGP/CMF+RTE, consumed enriched cereal-grain products/corn masa flour plus ready-to-eat cereals; ECGP/CMF+SUP, consumed enriched cereal-grain products/corn masa flour (excluding ready-to-eat cereals) plus supplements containing folic acid; ECGP/CMF+RTE+SUP, consumed enriched cereal-grain products/corn masa flour, ready-to-eat cereals, and supplements containing folic acid.

^10^Adjusted for age, race and FIPR

^11^ Categorized using Diabetes NHANES questionnaire, fasting blood sugar and glycohemoglobin level (HBA1c); adjusted for age, race, FIPR and BMI

Abbreviations: Body mass index (BMI), Family-income-poverty ratio (FIPR), Interquartile range (IQR)

**Supplemental Table 3A**

**Adjusted odds ratios for serum MeFox >90^th^ vs. ≤90^th^ percentile, non-pregnant women of reproductive age, 12–49 years: National Health and Nutrition Examination Survey 2011–March 2020**

|  | aOR (95% CI) | P value |
| --- | --- | --- |
| Glycohemoglobin (%) | 1.30 (1.16-1.46) | <0.0001 |
| Race/Ethnicity |  |  |
| Non-Hispanic Black | 0.40 (0.28-0.58) | <0.0001 |
| Non-Hispanic Asian | 0.62 (0.38-1.01) | 0.0529 |
| Hispanic^1^ | 0.37 (0.26-0.54) | <0.0001 |
| Non-Hispanic White | Reference | -- |
| Age^2^ | 1.01 (1.00-1.03) | 0.1112 |
| Body mass index (kg/m^2^) | 1.05 (1.03-1.06) | <0.0001 |
| Folic acid (μg/day) |  |  |
| ≥400 | 1.40 (0.94-2.07) | 0.0942 |
| < 400 | Reference |  |

^1^ “Hispanic” include respondents self-identified as “Mexican American” and self-identified “Hispanic” ethnicity. non-Hispanic (NH) participants were categorized based on their self-reported races.

^2^ Age in years

Sample size, n = 3626

Abbreviation: Pyrazino-s-triazine derivative of 4-alpha-hydroxy-5-methyltetrahydrofolate (MeFox)

**Supplemental Table 3B**

**Adjusted odds ratios for serum MeFox >90^th^ vs. ≤90^th^ percentile, non-pregnant women of reproductive age, 12–49 years: National Health and Nutrition Examination Survey 2011–March 2020**

|  | aOR (95% CI) | P value |
| --- | --- | --- |
| Diabetes status |  |  |
| Diabetes | 2.16 (1.34-3.46) | 0.0018 |
| Prediabetes | 1.06 (0.78-1.44) | 0.7040 |
| No diabetes | Reference | -- |
| Race/Ethnicity |  |  |
| Non-Hispanic Black | 0.44 (0.31-0.62) | <0.0001 |
| Non-Hispanic Asian | 0.64 (0.40-1.03) | 0.0665 |
| Hispanic^1^ | 0.38 (0.26-0.55) | <0.0001 |
| Non-Hispanic White | Reference | -- |
| Age^2^ | 1.01 (1.00-1.03) | 0.1162 |
| Body mass index (kg/m^2^) | 1.05 (1.03-1.06) | <0.0001 |
| Folic acid (μg/day) |  |  |
| ≥400 | 1.35 (0.92-1.98) | 0.1207 |
| < 400 | Reference |  |

^1^ “Hispanic” include respondents self-identified as “Mexican American” and self-identified “Hispanic” ethnicity. non-Hispanic (NH) participants were categorized based on their self-reported races.

^2^ Age in years

Sample size, n = 3626

Abbreviation: Pyrazino-s-triazine derivative of 4-alpha-hydroxy-5-methyltetrahydrofolate (MeFox)

**Supplemental Table 3C**

**Adjusted odds ratios for serum MeFox >90^th^ vs. ≤90^th^ percentile, non-pregnant women of reproductive age, 12–49 years: National Health and Nutrition Examination Survey 2011–March 2020**

|  | aOR (95% CI) | P value |
| --- | --- | --- |
| Diabetes status |  |  |
| Diabetes-Controlled^1^ | 0.82 (0.18-3.65) | 0.7902 |
| Diabetes-Uncontrolled^1^ | 2.36 (1.44-3.87) | 0.0009 |
| Prediabetes | 1.06 (0.78-1.44) | 0.7019 |
| No diabetes | Reference | -- |
| Race/Ethnicity |  |  |
| Non-Hispanic Black | 0.44 (0.31-0.62) | <0.0001 |
| Non-Hispanic Asian | 0.64 (0.40-1.03) | 0.0654 |
| Hispanic^2^ | 0.38 (0.26-0.55) | <0.0001 |
| Non-Hispanic White | Reference | -- |
| Age^3^ | 1.01 (1.00-1.03) | 0.1183 |
| Body mass index (kg/m^2^) | 1.05 (1.03-1.06) | <0.0001 |
| Folic acid (μg/day) |  |  |
| ≥400 | 1.37 (0.94-2.00) | 0.1026 |
| < 400 | Reference |  |

^1^ Diabetes: Controlled (HbA1c <5.7), Uncontrolled (HbA1c ≥5.7)

^2^ “Hispanic” include respondents self-identified as “Mexican American” and self-identified “Hispanic” ethnicity. non-Hispanic (NH) participants were categorized based on their self-reported races.

^3^ Age in years

Sample size, n = 3626

Abbreviation: Pyrazino-s-triazine derivative of 4-alpha-hydroxy-5-methyltetrahydrofolate (MeFox)

**Supplemental Table 4**

**Association of glycohemoglobin concentrations on folate status among non-pregnant women of reproductive age, 12–49 years: National Health and Nutrition Examination Survey 2011–March 2020**

|  | RBC folate (nmol/L) | | | | Serum MeFox (nmol/L) | | | |
| --- | --- | --- | --- | --- | --- | --- | --- | --- |
|  | N | Mean^1^ (95% CI) | aOR^2^ (95% CI) | P value | N | Mean^1^ (95% CI) | aOR^2^ (95% CI) | P value |
| HbA1c ≥5.7 vs. <5.7 | | | | | | | | |
| Among only those with self-reported diabetes | 151 |  |  |  | 150 |  |  |  |
| ≥5.7 | 132 | 1391 (1230-1574) | 7.66 (1.19-49.17) | 0.0324 | 131 | 1.59 (1.27-1.98) | 2.49 (1.20-5.18) | 0.0155 |
| <5.7 | 19 | 956 (689-1326) | Reference | -- | 19 | 1.16 (0.92-1.47) | Reference | -- |
| Among only those with diabetes (self-reported and undiagnosed) | 229 |  |  |  | 226 |  |  |  |
| ≥5.7 | 201 | 1337 (1216-1469) | 8.28 (2.29-29.94) | 0.0016 | 198 | 1.51 (1.28-1.78) | 2.61 (1.28-5.31) | 0.0089 |
| <5.7 | 28 | 942 (776-1144) | Reference | -- | 28 | 1.07 (0.89-1.30) | Reference | -- |
| Self-reported diabetes (HbA1c ≥5.7) vs. All WRA^3^ (HbA1c <5.7) | 2443 |  |  |  | 2408 |  |  |  |
| ≥5.7 | 132 | 1327 (1157-1523) | 3.96 (1.86-8.42) | 0.0005 | 131 | 1.44 (1.15-1.81) | 2.30 (1.59-3.31) | <0.0001 |
| <5.7 | 2311 | 1034 (1010-1059) | Reference | -- | 2277 | 0.99 (0.96-1.03) | Reference | -- |
| All those with diabetes (HbA1c ≥5.7) vs. All WRA^3^ (HbA1c <5.7) | 2521 |  |  |  | 2484 |  |  |  |
| ≥5.7 | 201 | 1279 (1154-1417) | 3.85 (1.98-7.48) | 0.0001 | 198 | 1.36 (1.16-1.60) | 2.24 (1.57-3.20) | <0.0001 |
| <5.7 | 2320 | 1035 (1011-1059) | Reference | -- | 2286 | 1.00 (0.96-1.04) | Reference | -- |
| All WRA, (HbA1c ≥5.7 vs. <5.7) | 3731 |  |  |  | 3677 |  |  |  |
| ≥5.7 | 678 | 1115 (1066-1166) | 1.52 (1.08-2.12) | 0.0166 | 667 | 1.19 (1.09-1.30) | 1.63 (1.29-2.06) | 0.0001 |
| <5.7 | 3053 | 1035 (1013-1058) | Reference | -- | 3010 | 1.00 (0.97-1.04) | Reference | -- |
|  |  | | |  |  |  |  |  |
| HbA1c ≥5.7 to <6.5 to <5.7 | | | | | | | | |
| Among only those with self-reported diabetes | 56 |  |  |  | 56 |  |  |  |
| ≥5.7 to <6.5 | 37 | 1385 (1165-1646) | 6.15 (0.93-40.65) | 0.059 | 37 | 1.49 (1.21-1.82) | 2.76 (0.69-11.04) | 0.1482 |
| <5.7 | 19 | 1061 (765-1471) | Reference | -- | 19 | 1.23 (1.05-1.45) | Reference | -- |
| Among only those with diabetes (self-reported and undiagnosed) | 82 |  |  |  | 81 |  |  |  |
| ≥5.7 to <6.5 | 54 | 1422 (1235-1637) | 12.92 (2.40-69.67) | 0.0034 | 53 | 1.55 (1.26-1.90) | 3.54 (1.09-11.49) | 0.0357 |
| <5.7 | 28 | 968 (777-1207) | Reference | -- | 28 | 1.11 (0.98-1.26) | Reference | -- |
| Self-reported diabetes (HbA1c ≥5.7 to <6.5) vs.  All WRA^3^ (HbA1c <5.7) | 2348 |  |  |  | 2314 |  |  |  |
| ≥5.7 to <6.5 | 37 | 1319 (1052-1654) | 4.07 (1.06-15.64) | 0.0414 | 37 | 1.32 (1.01-1.72) | 2.46 (1.13-5.34) | 0.0236 |
| <5.7 | 2311 | 1033 (1009-1058) | Reference | -- | 2277 | 0.99 (0.96-1.03) | Reference | -- |
| All those with diabetes (HbA1c ≥5.7 to <6.5) vs.  All WRA^3^ (HbA1c <5.7) | 2374 |  |  |  | 2339 |  |  |  |
| ≥5.7 to <6.5 | 54 | 1314 (1113-1552) | 5.22 (1.65-16.47) | 0.0054 | 53 | 1.35 (1.07-1.70) | 2.67 (1.35-5.26) | 0.0052 |
| <5.7 | 2320 | 1034 (1010-1058) | Reference | -- | 2286 | 0.99 (0.96-1.03) | Reference | -- |
| All WRA, (HbA1c ≥5.7 to <6.5 vs. <5.7) | 3584 |  |  |  | 3532 |  |  |  |
| ≥5.7 to <6.5 | 531 | 1082 (1030-1138) | 1.28 (0.88-1.87) | 0.1876 | 522 | 1.14 (1.05-1.24) | 1.53 (1.18-1.98) | 0.0018 |
| <5.7 | 3053 | 1034 (1012-1057) | Reference | -- | 3010 | 1.00 (0.97-1.04) | Reference | -- |
|  | | | |  |  |  |  |  |
| HbA1c ≥6.5 vs. <5.7 | | | | | | | | |
| Among only those with self-reported diabetes | 114 |  |  |  | 113 |  |  |  |
| ≥6.5 | 95 | 1374 (1171-1612) | 7.63 (1.37-42.43) | 0.0209 | 94 | 1.64 (1.21-2.23) | 2.37 (1.08-5.18) | 0.0317 |
| <5.7 | 19 | 910 (662-1250) | Reference | -- | 19 | 1.14 (0.87-1.49) | Reference | -- |
| Among only those with diabetes (self-reported and undiagnosed) | 175 |  |  |  | 173 |  |  |  |
| ≥6.5 | 147 | 1300 (1151-1467) | 6.94 (2.06-23.39) | 0.0022 | 145 | 1.51 (1.21-1.88) | 2.41 (1.13-5.16) | 0.0236 |
| <5.7 | 28 | 921 (767-1104) | Reference | -- | 28 | 1.07 (0.88-1.29) | Reference | -- |
| Self-reported diabetes (HbA1c ≥6.5) vs. All WRA^3^ (HbA1c <5.7) | 2406 |  |  |  | 2371 |  |  |  |
| ≥6.5 | 95 | 1330 (1116-1585) | 4.24 (1.73-10.38) | 0.002 | 94 | 1.51 (1.09-2.10) | 2.29 (1.56-3.37) | 0.0001 |
| <5.7 | 2311 | 1033 (1008-1057) | Reference | -- | 2277 | 0.99 (0.96-1.03) | Reference | -- |
| All those with diabetes (HbA1c ≥6.5) vs. All WRA^3^ (HbA1c <5.7) | 2467 |  |  |  | 2431 |  |  |  |
| ≥6.5 | 147 | 1257 (1107-1428) | 3.47 (1.63-7.39) | 0.0015 | 145 | 1.36 (1.09-1.69) | 2.11 (1.50-2.96) | <0.0001 |
| <5.7 | 2320 | 1033 (1009-1058) | Reference | -- | 2286 | 1.00 (0.96-1.03) | Reference | -- |
| All WRA, (HbA1c ≥6.5 vs. <5.7) | 3200 |  |  |  | 3155 |  |  |  |
| ≥6.5 | 147 | 1279 (1126-1452) | 3.74 (1.79-7.78) | 0.0006 | 145 | 1.40 (1.12-1.76) | 2.21 (1.58-3.10) | <0.0001 |
| <5.7 | 3053 | 1036 (1014-1059) | Reference | -- | 3010 | 1.00 (0.97-1.04) | Reference | -- |
|  | | | |  |  |  |  |  |
| HbA1c ≥7 vs. <5.7 | | | | | | | | |
| Among only those with self-reported diabetes | 95 |  |  |  | 94 |  |  |  |
| ≥7 | 76 | 1281 (1144-1434) | 8.13 (1.14-58.07) | 0.0371 | 75 | 1.36 (1.17-1.58) | 2.00 (0.86-4.62) | 0.1044 |
| <5.7 | 19 | 909 (690-1198) | Reference | -- | 19 | 1.07 (0.86-1.32) | Reference | -- |
| Among only those with diabetes (self-reported and undiagnosed) | 131 |  |  |  | 129 |  |  |  |
| ≥7 | 103 | 1244 (1120-1382) | 7.65 (1.87-31.35) | 0.0053 | 101 | 1.32 (1.15-1.52) | 2.51 (1.07-5.90) | 0.0352 |
| <5.7 | 28 | 929 (786-1098) | Reference | -- | 28 | 1.04 (0.91-1.19) | Reference | -- |
| Self-reported diabetes (HbA1c ≥7) vs. All WRA^3^ (HbA1c <5.7) | 2387 |  |  |  | 2352 |  |  |  |
| ≥7 | 76 | 1239 (1060-1448) | 3.28 (1.16-9.27) | 0.0255 | 75 | 1.24 (1.06-1.46) | 1.83 (1.14-2.94) | 0.0136 |
| <5.7 | 2311 | 1032 (1008-1057) | Reference | -- | 2277 | 0.99 (0.96-1.03) | Reference | -- |
| All those with diabetes (HbA1c ≥7) vs. All WRA^3^ (HbA1c <5.7) | 2423 |  |  |  | 2387 |  |  |  |
| ≥7 | 103 | 1206 (1064-1366) | 3.11 (1.27-7.64) | 0.0141 | 101 | 1.19 (1.03-1.38) | 1.80 (1.15-2.82) | 0.0107 |
| <5.7 | 2320 | 1032 (1008-1057) | Reference | -- | 2286 | 0.99 (0.96-1.03) | Reference | -- |
| All WRA, (HbA1c ≥7 vs. <5.7) | 3156 |  |  |  | 3111 |  |  |  |
| ≥7 | 103 | 1224 (1085-1381) | 3.21 (1.36-7.55) | 0.0084 | 101 | 1.23 (1.06-1.44) | 1.85 (1.19-2.89) | 0.0074 |
| <5.7 | 3053 | 1036 (1014-1059) | Reference | -- | 3010 | 1.00 (0.96-1.04) | Reference | -- |

^1^ Adjusted for age, race, body mass index, family-income-poverty ratio, smoking status, eGFR and supplement use

^2^ Adjusted for age, race, body mass index, eGFR and supplement use

^3^ Those with controlled diabetes and those without diabetes (excludes participants with prediabetes)

Abbreviations: Estimated glomerular filtration rate (eGFR), Pyrazino-s-triazine derivative of 4-alpha-hydroxy-5-methyltetrahydrofolate (MeFox), Red blood cell (RBC), Women of reproductive age (WRA)

**Supplemental Table 5**

**Adjusted RBC folate concentrations and medication use among non-pregnant women of reproductive age 12–49 years with diabetes: National Health and Nutrition Examination Survey 2011–March 2020**

|  | RBC folate (nmol/L) | | | | | | | |
| --- | --- | --- | --- | --- | --- | --- | --- | --- |
| Medication use | Overall^1^  (n = 229) | | Diagnosed^2^  (n = 151) | | Diagnosed (HbA1c <6.5)^3^  (n = 56) | | Undiagnosed^1^  (n = 78) | |
|  | n | Mean^4^ (95% CI) | n | Mean^4^ (95% CI) | n | Mean^4^ (95% CI) | n | Mean^4^ (95% CI) |
| Type of diabetic medication |  | | | | | | | |
| No metformin or insulin | 128 | 1112 (1020-1212) | 55 | 1049 (900-1222) | 31 | 1096 (997-1270) | 73 | 1157 (900-12223) |
| Metformin only | 60 | 1638 (1375-1952) | 55 | 1632 (1338-1990) | 21 | 1518 (1230-1873) | 5 | 1727 (1226-2432) |
| Metformin and insulin | 19 | 1420 (962-2096) | 19 | 1420 (962-2096) | 2 | -- | 0 | -- |
| Insulin only | 22 | 1436 (1127-1830) | 22 | 1436 (1127-1830) | 2 | -- | 0 | -- |
| Number of prescriptions |  | | | | | | | |
| <5 | 171 | 1193 (1074-1325) | 100 | 1474 (1117-1945) | 63 | 1103 (962-1265) | 71 | 1158 (1026-1306) |
| ≥5 | 58 | 1602 (1390-1846) | 51 | 1620 (1400-1945) | 19 | 1815 (1498-2199) | 7 | 1474 (1117-1945) |

^1^ Overall (n = 229): categorized using Diabetes NHANES questionnaire, fasting blood sugar and glycohemoglobin level (HbA1c); includes self-reported (n = 151) and undiagnosed (n = 78)

^2^ Diagnosed (n = 151): includes only those who self-reported to having diabetes

^3^ Diagnosed (HbA1c), (n = 56, [41.8%, 95% CI: 32.1–52.2] ): includes only those who self-reported to having diabetes with HbA1c <6.5

^4^ Geometric Mean: Adjusted for age, race, body mass index, estimated glomerular filtration rate, family-income-poverty ratio, smoking exposure and supplement use

Abbreviation: Red blood cell (RBC)


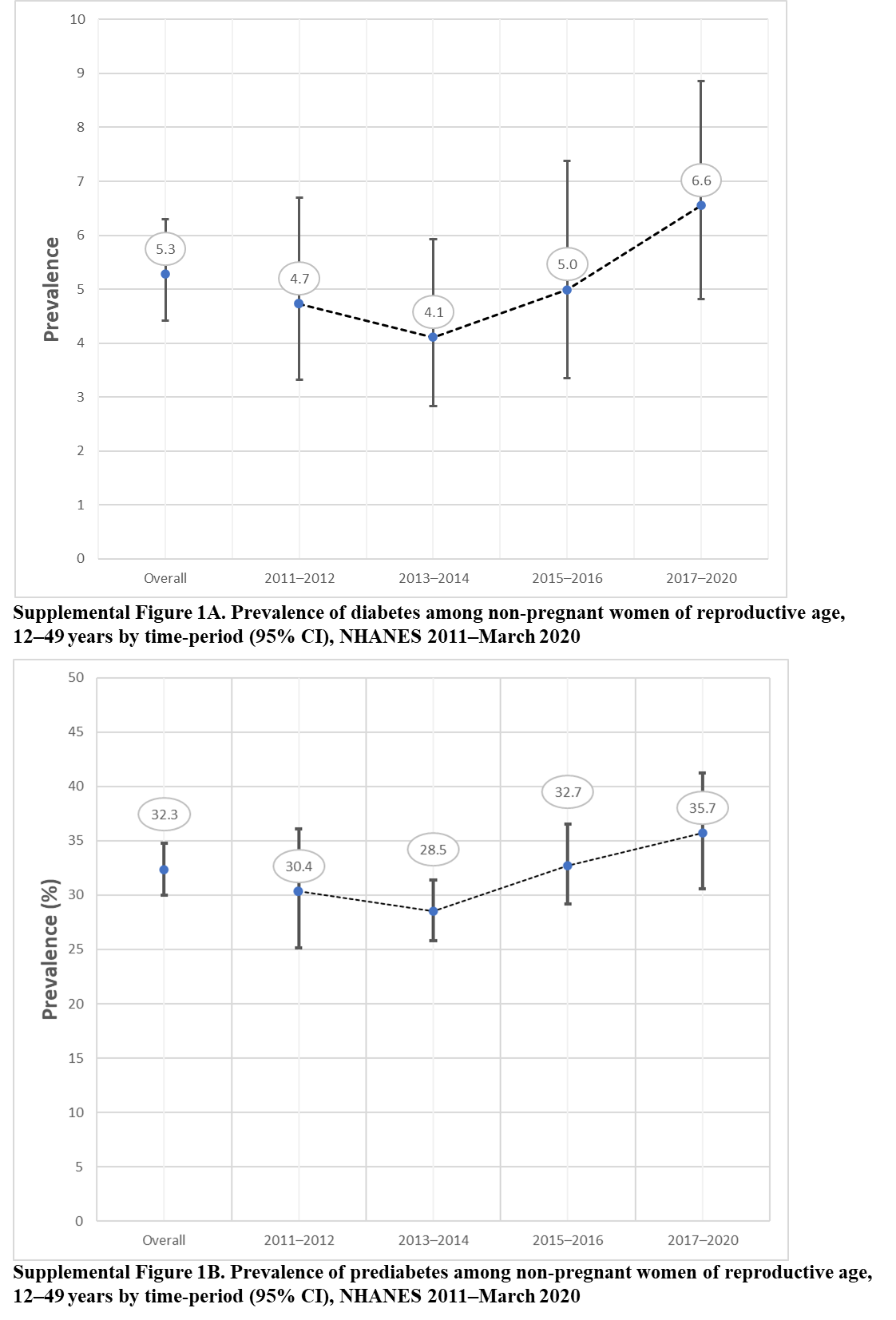
 **Supplemental Figures 1 (A-B)**

**Supplemental Figure 1A.** **Prevalence of diabetes among non-pregnant women of reproductive age, 12–49 years by time-period (95% CI): NHANES 2011–March 2020.** Overall weighted percent of diabetes by NHANES cycles (2011–2012, 2013–2014, 2015–2016 and 2017–2020). Two-sided Wald 2011–2012 vs. 2017–2020 p = 0.16, p = 0.12 trend 2011–2020.

**Supplemental Figure 1B.** **Prevalence of prediabetes among non-pregnant women of reproductive age, 12–49 years by time-period (95% CI): NHANES 2011–March 2020.** Overall weighted percent of prediabetes by NHANES cycles (2011–2012, 2013–2014, 2015–2016 and 2017–2020). Two-sided Wald 2011–2012 vs. 2017–2020 p = 0.16, p = 0.08 trend 2011–2020.


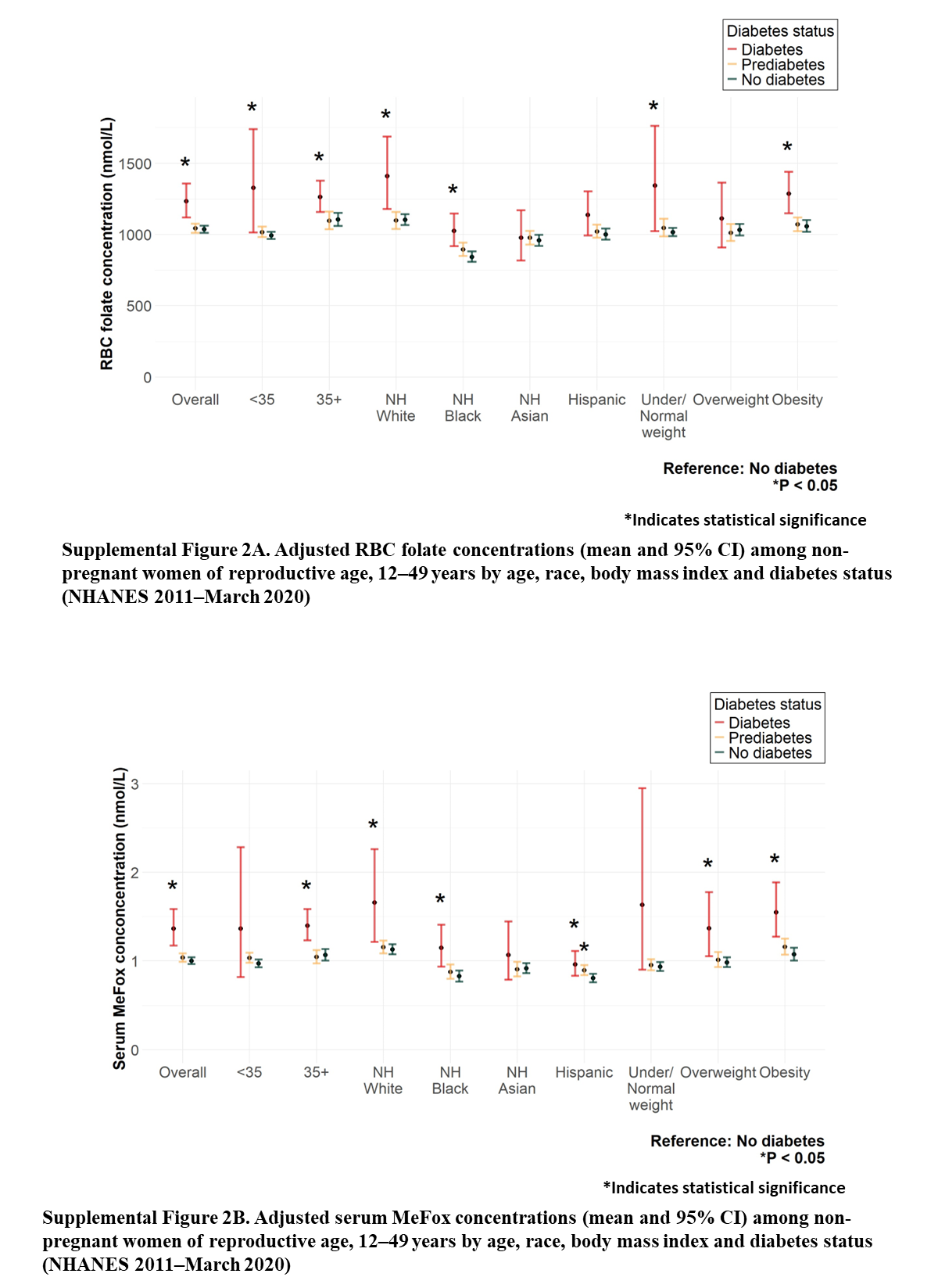


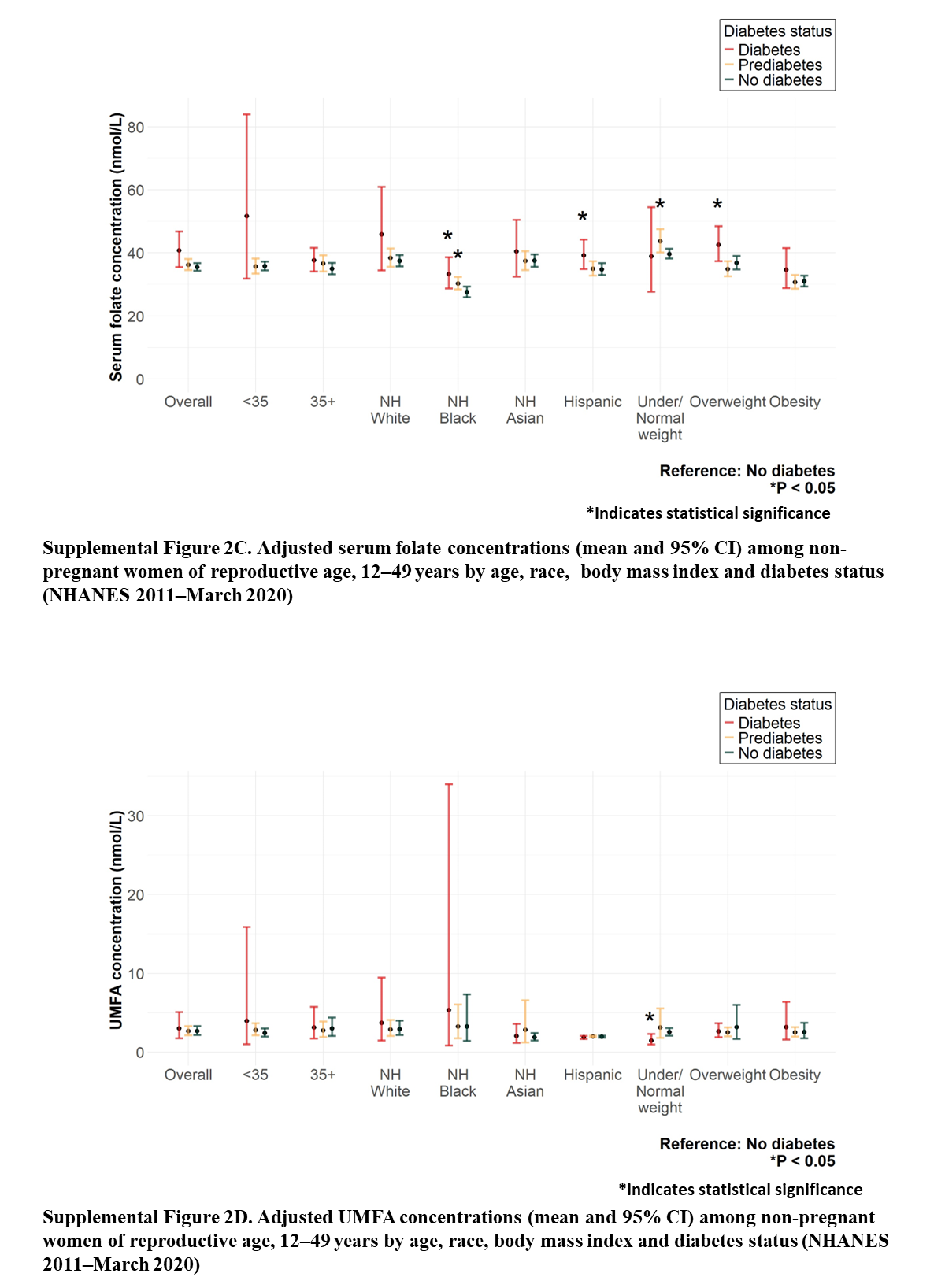


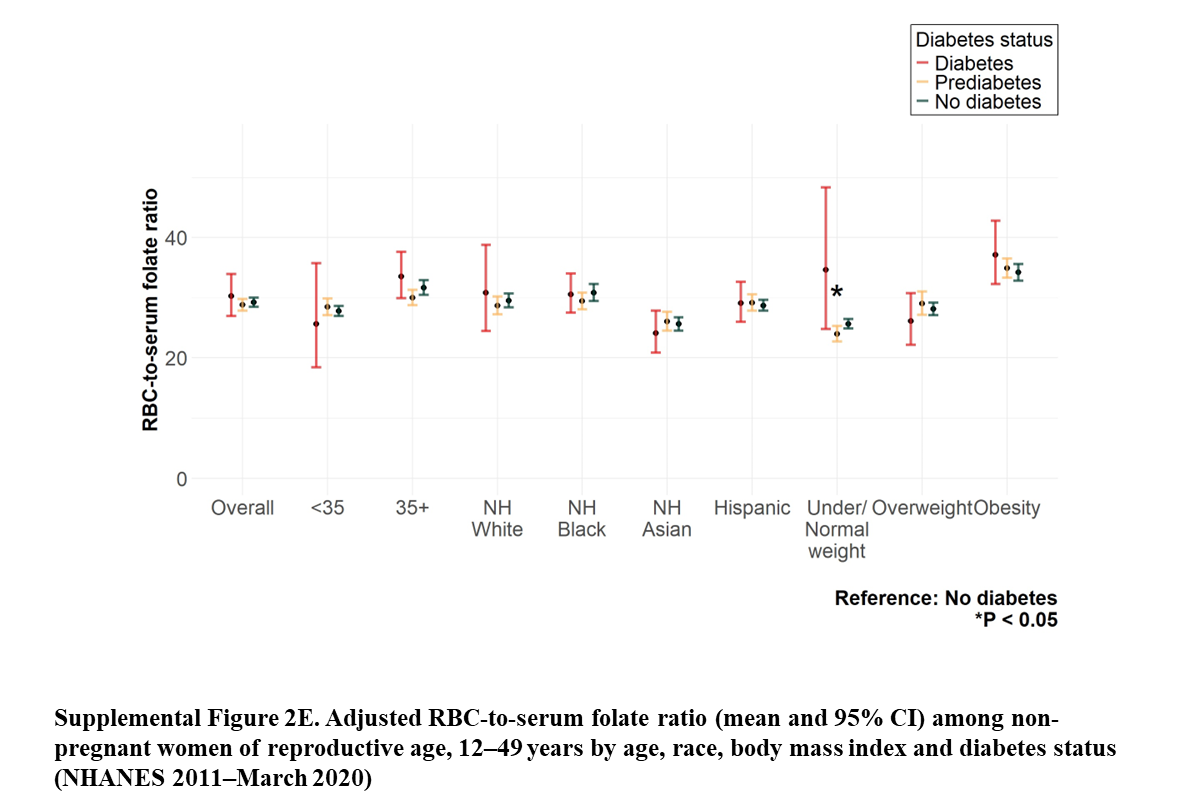


**Figure 2A. Adjusted RBC folate concentrations (mean and 95% CI) among non-pregnant women of reproductive age, 12–49 years by age, race, body mass index and diabetes status: NHANES 2011–March 2020.** RBC folate concentrations were higher among participants with diabetes compared to those without diabetes. Differences in RBC folate concentrations between participants with diabetes to those without diabetes were significant overall^1^, and for the following subgroups: ≥35y^2^, non-Hispanic White^3^, non-Hispanic Black^4^ , under/normal weight^4^ and obesity^4^ .

**Supplemental Figure 2B. Adjusted serum MeFox concentrations (mean and 95% CI) among non-pregnant women of reproductive age, 12–49 years by age, race, body mass index and diabetes status: NHANES 2011–March 2020.** Serum MeFox concentrations were higher among participants with diabetes compared to those without diabetes. Serum MeFox concentrations for overall^1^, ≥35y^2^, non-Hispanic White^3^, non-Hispanic Black^3^, Hispanic^3^, overweight^4^ and obesity^4^ were statistically significant comparing participants with diabetes to those without diabetes.

^1^ Adjusted for age, race, body mass index, estimated glomerular filtration rate, family-income-poverty ratio income ratio, smoking exposure, supplement use

^2^ Adjusted for race, body mass index, estimated glomerular filtration rate, family-income-poverty ratio income ratio, smoking exposure, supplement use

^3^ Adjusted for age, body mass index, estimated glomerular filtration rate, family-income-poverty ratio income ratio, smoking exposure, supplement use

^4^ Adjusted for age, race, estimated glomerular filtration rate, family-income-poverty ratio income ratio, smoking exposure, supplement use

Abbreviations: non-Hispanic (NH), pyrazino-s-triazine derivative of 4-α-hydroxy-5-methyltetrahydrofolate (MeFox), Red blood cell (RBC)

**Supplemental Figure 2C. Adjusted serum concentrations (mean and 95% CI) among non-pregnant women of reproductive age, 12–49 years by age, race, body mass index and diabetes status: NHANES 2011–March 2020.** Except for underweight, serum folate concentration was higher among participants with diabetes compared to those without diabetes. Serum folate concentrations for non-Hispanic Black^1^, Hispanic^1^ and overweight^2^ were statistically significant comparing participants with diabetes to those without diabetes. **Supplemental Figure 2D. Adjusted UMFA concentrations (mean and 95% CI) among non-pregnant women of reproductive age, 12–49 years by age, race, body mass index and diabetes status: NHANES 2011–March 2020.** For underweight, UMFA concentration was lower and statistically significant comparing participants with diabetes to those without diabetes. UMFA concentrations for overall^3^, <35y^4^, ≥35y^4^, non-Hispanic White^1^, non-Hispanic Black^1^ , non-Hispanic Asian^1^, Hispanic^1^, overweight^2^ and obesity^2^ were not statistically significant. **Supplemental Figure 2E. Adjusted RBC-to-serum ratio (mean and 95% CI) among non-pregnant women of reproductive age, 12–49 years by age, race, body mass index and diabetes status: NHANES 2011–March 2020.** RBC-to-serum ratio for overall^3^, <35y^4^, ≥35y^4^, non-Hispanic White^1^, non-Hispanic Black^1^ , non-Hispanic Asian^1^, Hispanic^1^, under/normal weight^2^, overweight^2^ and obesity^2^ were not statistically significant comparing participants with diabetes to those without diabetes.

^1^ Adjusted for age, body mass index, estimated glomerular filtration rate, family-income-poverty ratio, smoking exposure, supplement use

^2^ Adjusted for age, race, estimated glomerular filtration rate, family-income-poverty ratio, smoking exposure, supplement use

^3^ Adjusted for age, race, body mass index, estimated glomerular filtration rate, family-income-poverty ratio, smoking exposure, supplement use

^4^ Adjusted for race, body mass index, estimated glomerular filtration rate, family-income-poverty ratio, smoking exposure, supplement use

Abbreviations: non-Hispanic (NH), Red blood cell (RBC), Unmetabolized folic acid (UMFA)


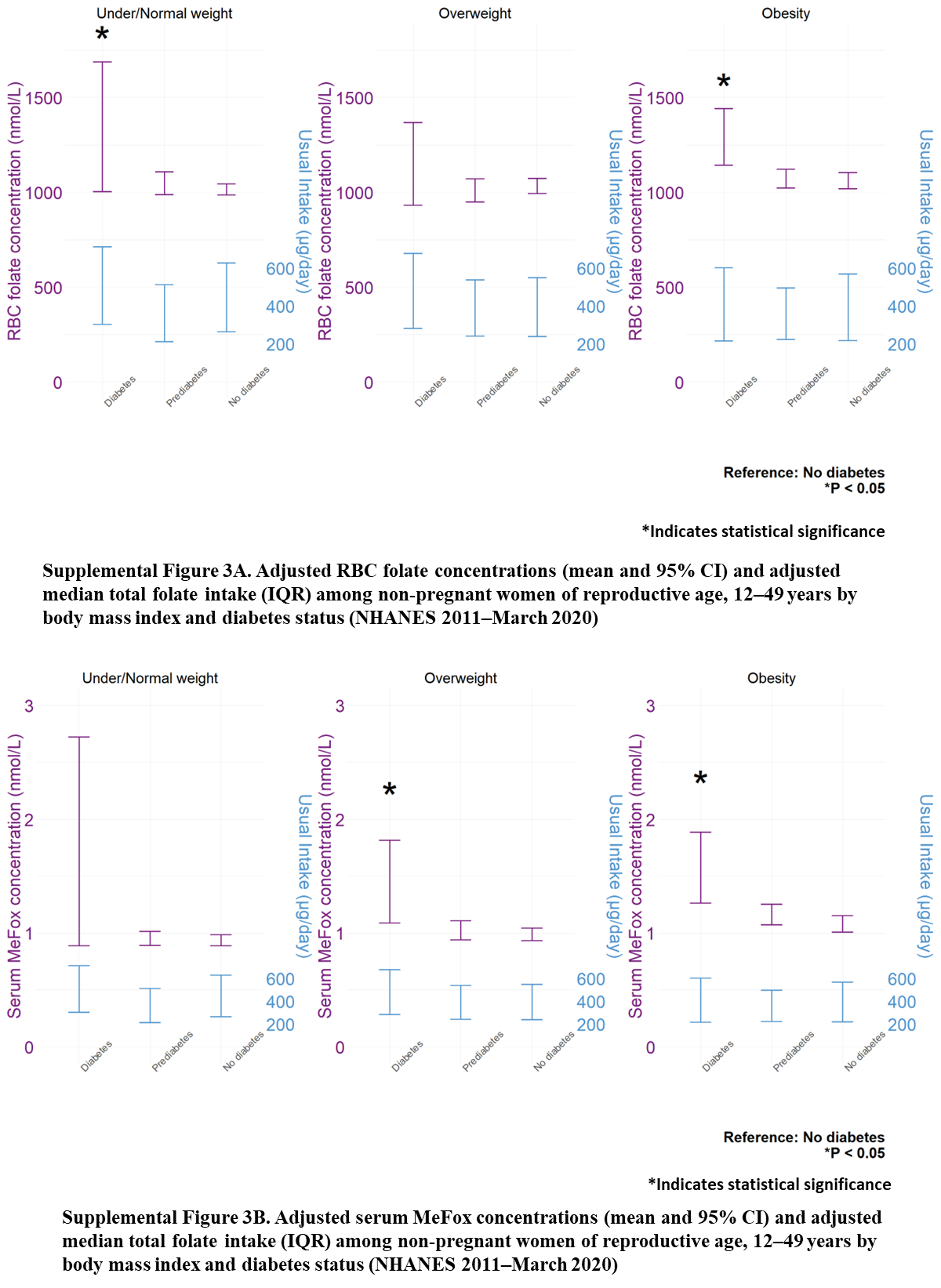


**Supplemental Figure 3A. Adjusted RBC folate concentrations (mean and 95% CI) and median total folate intake (IQR) among non-pregnant women of reproductive age, 12–49 years by diabetes status and body mass index: NHANES 2011–March 2020.** RBC folate concentration^1^ among under/normal weight participants and those with obesity were higher and statistically significant comparing those with diabetes to those without diabetes, with no difference in median folic acid intake^2, 3^ (μg/day).

**Supplemental Figure 3B. Adjusted serum MeFox concentrations (mean and 95% CI) and median total folate intake (IQR) among non-pregnant women of reproductive age, 12–49 years by diabetes status and body mass index: NHANES 2011–March 2020.** Serum MeFox concentrations^1^ among overweight participants and those with obesity was higher and statistically significantly comparing those with diabetes to those without diabetes, with no difference in median folic acid intake^2, 3^ (μg/day).

^1^ Adjusted for age, race, body mass index, estimated glomerular filtration rate, family-income-poverty ratio, smoking exposure, supplement use

^2^ Median intake and IQR accounting for the complex sampling design, adjustment for variations in day 1, day 2 measurements and by the day of the week; statistical analyses performed using Monte Carlo simulated pseudo-persons (each participant generated 100 pseudo-persons of the participant)

^3^ Adjusted for adjusted for age, race, family-income-poverty ratio and body mass index

Abbreviations: Interquartile range (IQR), Pyrazino-s-triazine derivative of 4-α-hydroxy-5-methyltetrahydrofolate (MeFox), Red blood cell (RBC)

Pink line: RBC folate and serum MeFox concentrations (nmol/L); Blue line: usual intake (μg/day)


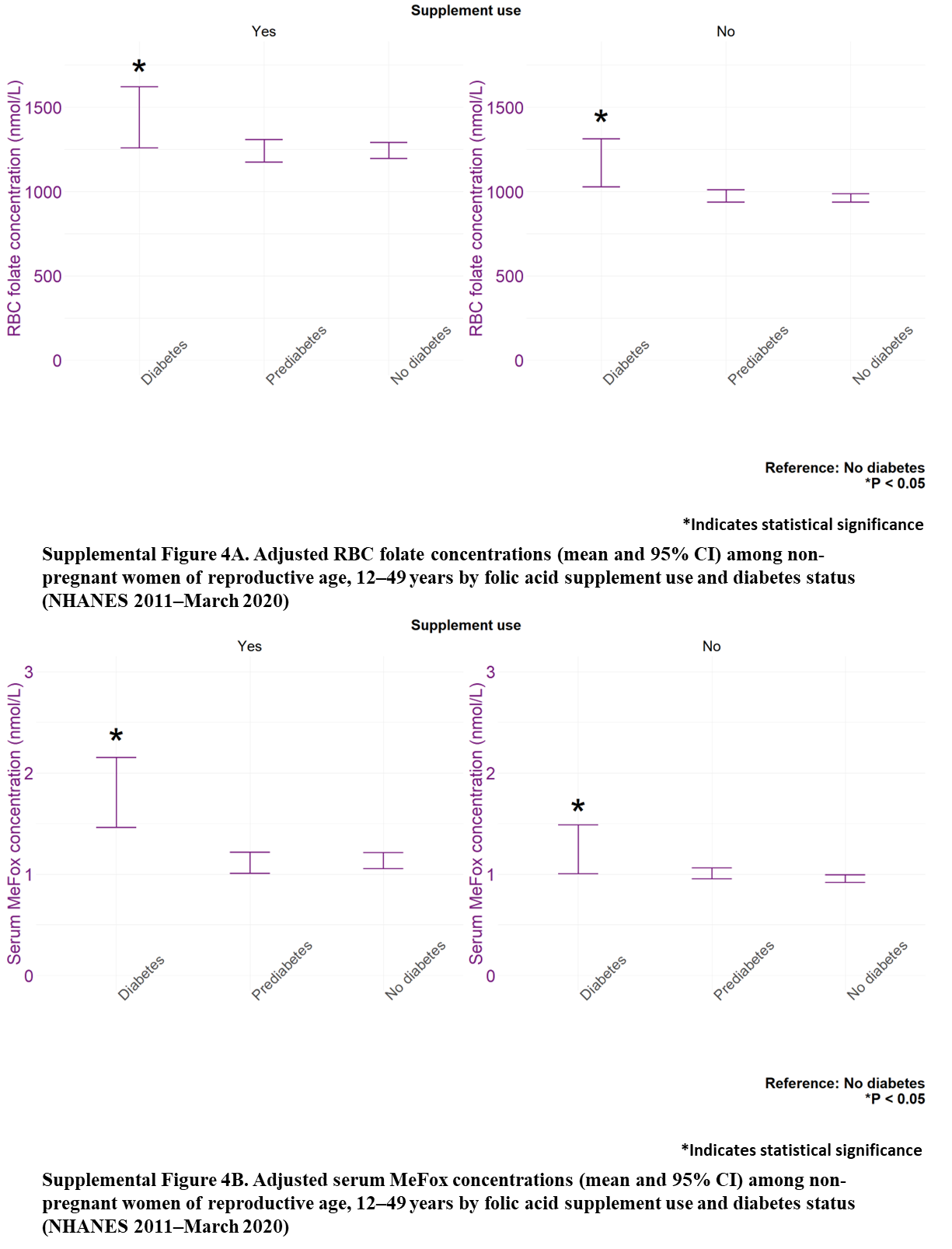


**Supplemental Figure 4A. Adjusted RBC folate concentrations (mean and 95% CI) among non-pregnant women of reproductive age, 12–49 years by folic acid supplement use and diabetes status: NHANES 2011–March 2020.** RBC folate concentration^1^ was higher and statistically significant among participants with diabetes compared to those without diabetes, regardless of folic acid supplementation status.

**Figure Supplemental 4B. Adjusted serum MeFox concentrations (mean and 95% CI) among non-pregnant women of reproductive age, 12–49 years by folic acid supplement use and diabetes status: NHANES 2011–March 2020.** Serum MeFox concentration^1^ was higher and statistically significant among participants with diabetes compared to those without diabetes, regardless of folic acid supplementation status.

^1^ Adjusted for age, race, body mass index, estimated glomerular filtration rate, family-income-poverty ratio and smoking exposure


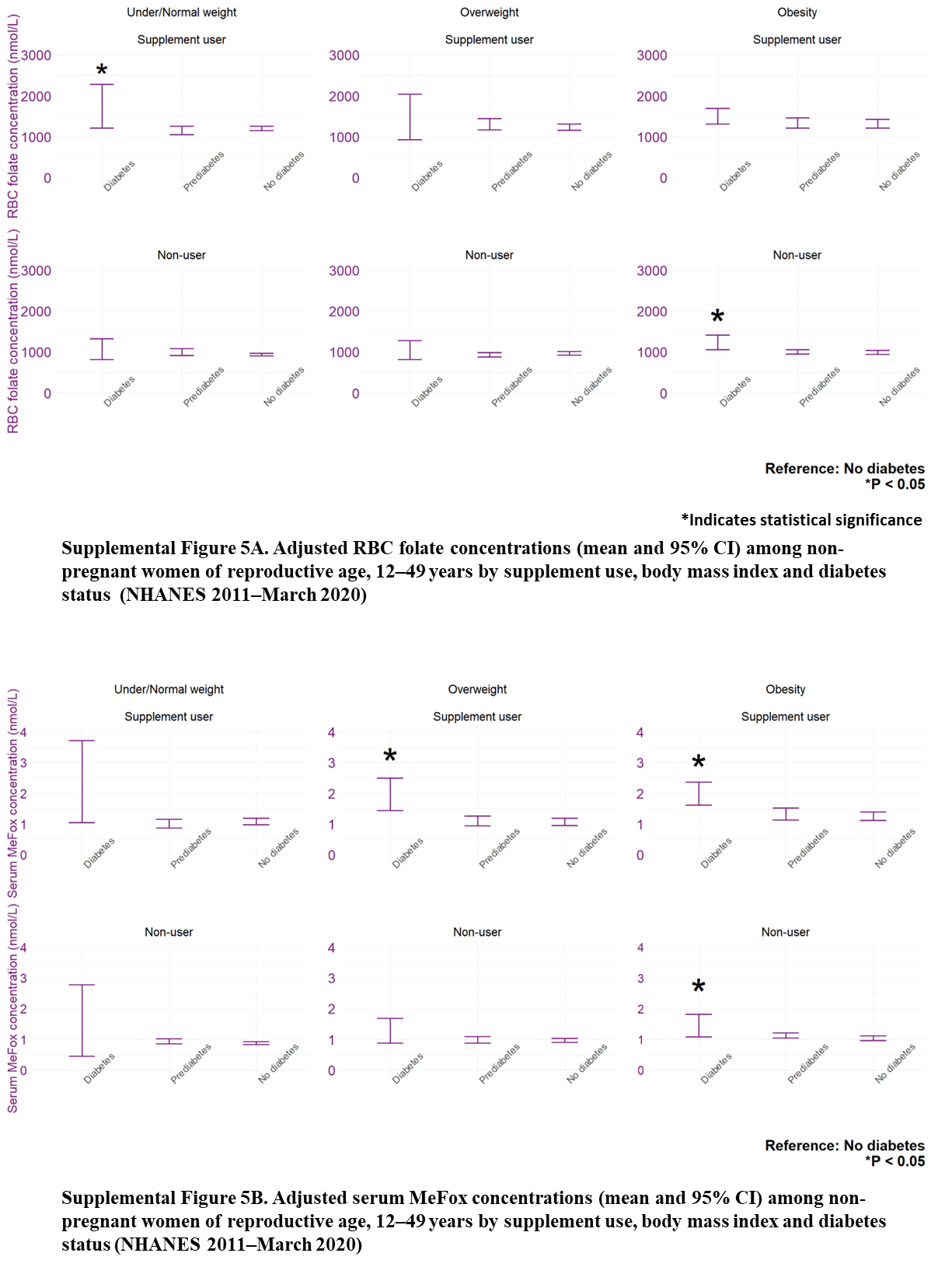


**Supplemental Figures 5 (A-B)**

**Supplemental Figure 5A. Adjusted RBC folate concentrations (mean and 95% CI) among non-pregnant women of reproductive age, 12–49 years by supplement use, body mass index and diabetes status: NHANES 2011–March 2020.** For under/normal weight participants taking folic acid supplements and those with obesity not taking folic acid supplementation, RBC folate concentration^1^ were higher and statistically significant comparing those with diabetes to those without diabetes.

**Supplemental Figure 5B. Adjusted serum MeFox concentrations (mean and 95% CI) among non-pregnant women of reproductive age, 12–49 years by folic acid supplement use, body mass index and diabetes status: NHANES 2011–March 2020.** For participants with obesity, serum MeFox concentration^1^ was higher and statistically significant comparing those with diabetes to those without diabetes, regardless of folic acid supplementation status.

^1^ Adjusted for age, race, estimated glomerular filtration rate, family-income-poverty ratio and smoking exposure

Abbreviations: Pyrazino-s-triazine derivative of 4-α-hydroxy-5-methyltetrahydrofolate (MeFox), Red blood cell (RBC)
